# *N*-Acetylneuraminic acid triggers endothelial pyroptosis and promotes atherosclerosis progression via GLS2-mediated glutaminolysis pathway

**DOI:** 10.1038/s41420-024-02233-7

**Published:** 2024-11-13

**Authors:** Zhaohong Liu, Peng Xiang, Shengmei Zeng, Ping Weng, Yilin Wen, Wanping Zhang, Hui Hu, Dezhang Zhao, Limei Ma, Chao Yu

**Affiliations:** 1 College of Pharmacy, Chongqing Medical University, Chongqing, China; 2 Chongqing Key Laboratory for Pharmaceutical Metabolism Research, Chongqing, China; 3 Key Laboratory for Biochemistry and Molecular Pharmacology of Chongqing, Chongqing, China; 4 Research Center for Innovative Pharmaceutical and Experiment Analysis Technology, Chongqing, China

**Keywords:** Cell biology, Diseases

## Abstract

Vascular endothelial injury initiates atherosclerosis (AS) progression. *N*-Acetylneuraminic acid (Neu5Ac) metabolic disorder was found to intensify endothelial mitochondrial damage. And GLS2-associated glutaminolysis disorder contributed to mitochondrial dysfunction. However, mechanisms underlying Neu5Ac-associated mitochondrial dysfunction as well as its association with GLS2 remains unclear. In this study, we constructed GLS2^−/−^ApoE^−/−^ mice by using HBLV-GLS2 shRNA injection. And methods like immunofluorescence, western blotting, transmission electron microscopy were applied to detect profiles of endothelial injury and AS progression both in vivo and in vitro. We demonstrated that Neu5Ac accumulation increased GLS2 expression and promoted glutaminolysis disorder, which further induced endothelial mitochondrial dysfunction via a pyroptosis-dependent pathway in vivo and in vitro. Mechanically, Neu5Ac interacted with SIRT3 and led to FOXO3a deacetylation and phosphorylation, further facilitated c-Myc antagonism and ultimately increased GLS2 levels. Inhibition of GLS2 could improve mitochondrial function and mitigate pyroptosis process. In addition, blocking Neu5Ac production using neuraminidases (NEUs) inhibitor could rescue endothelial damage and alleviate AS development in ApoE^−/−^ mice. These findings proposed that Neu5Ac induced GLS2-mediated glutaminolysis disorder and then promoted mitochondrial dysfunction in a pyroptosis-dependent pathway. Targeting GLS2 or inhibiting Neu5Ac production could prevent AS progression.

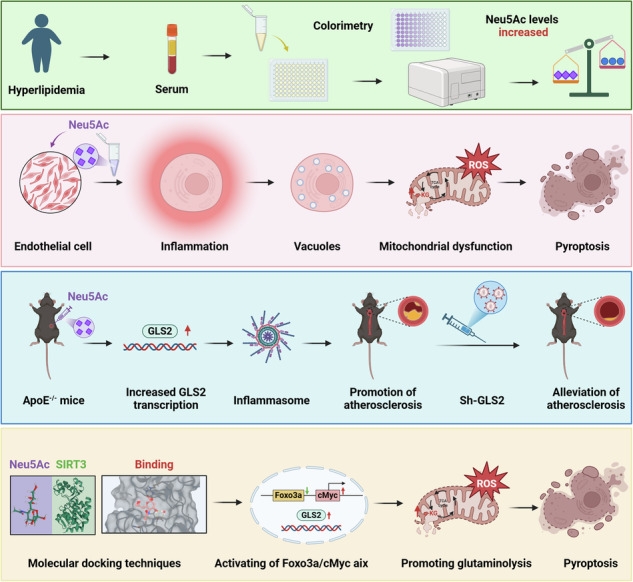

## Background

Atherosclerotic cardiovascular disease (ASCVD) remains to be the leading cause of global mortality and morbidity [1, 2]. Initiating factors for the progression of ASCVD includes endothelial dysfunction and vascular inflammation [3]. Recent evidence highlights various metabolic biomarkers which may play significant roles in ASCVD development, particularly emphasizes metabolites in the peripheral circulation [4–10]. However, mechanisms governing the regulation of metabolites in maintaining vascular homeostasis have not been fully elucidated.

*N*-Acetylneuraminic acid (Neu5Ac) is commonly situated at the terminus of oligosaccharide chains present in glycoproteins and glycolipids [11]. Epidemiological investigations have indicated that heightened concentration of Neu5Ac functions as a notable metabolic biomarker for ASCVD progression [11–13]. Our prior study also proved above epidemiological investigations in ApoE^−/^^−^ mice. And the results further indicated that Neu5Ac exacerbated atherosclerosis (AS) progression partly through endothelial ferroptosis pathway. Particularly, mitochondrial dysfunction was strongly involved in AS progression induced by Neu5Ac [14, 15], which raises the possibility of preventing mitochondrial damage induced by Neu5Ac might be a potential therapeutic target for ASCVD treatment. However, the precise mechanism underlying Neu5Ac-associated mitochondrial dysfunction remains incompletely understood, necessitating further investigation.

Pyroptosis is a gasdermin-mediated programmed cell death process associated with inflammasome activation and then release numerous pro-inflammatory factors [16]. Researchers have confirmed that pyroptosis activation strongly participated in AS progression [17]. Specially, endothelial cell (EC) pyroptosis leads to endothelial dysfunction through NLRP3 inflammasome assembly and Caspase-1 activation, thereby initiating AS progression [18–20]. Furthermore, EC pyroptosis activation was found to be highly linked to mitochondrial damage and ROS production [21–23]. Therefore, ROS/NLRP3 axis plays a critical role both in EC pyroptosis activation and initiation of endothelial injury. Our previous study have found that Neu5Ac possessed potential ability to induce mitochondrial damage and triggered ROS generation [14, 15]. However, its capacity to promote EC pyroptosis as well as underlying mechanism remains unexplored.

Glutaminase 2 (GLS2) is a glutaminolysis enzyme, catalyzing the conversion of glutamine (Gln) to glutamate (Glu) and further toward to the generation of factors including adenosine triphosphate (ATP) or ROS or glutathione (GSH) [24, 25]. Researchers have found that GLS2 could be activated in AS progression [26]. And Zhou et al. demonstrated that upregulation of GLS2 could induce the increasing of ROS, malondialdehyde (MDA) and iron ions (Fe^2+^), which further initiated ferroptosis pathway [27]. These results indicated that GLS2 should be involved in ferroptosis pathway. Our previous study have pointed out the association between Neu5Ac disruption and ferroptosis activation. We also identified the mitochondrial dysfunction as a contributing factor to ferroptosis activation [14]. Nevertheless, the specific involvement of GLS2 in Neu5Ac-related mitochondrial dysfunction as well as its association with pyroptosis in AS process remains inadequately understood.

To further explore the mechanisms underlying Neu5Ac-associated endothelial injury in AS process, we replicated the endothelial dysfunction models as reported before [14]. We found that Neu5Ac increased GLS2 levels and further induced mitochondrial dysfunction, then triggered endothelial pyroptosis through SIRT3/FOXO3a-c-MYC signaling pathway. Additionally, we found that inhibition of GLS2 or blocking Neu5Ac production could recuse arterial cell viability and mitigate AS progression. Taken together, our present study further elucidated the regulation mechanism between GLS2 and Neu5Ac, and also present a promising intervention strategy for ASCVD treatment.

## Results

### Neu5Ac contributed to endothelial inflammatory injury through NLRP3 dependent pyroptosis

**Fig. 1:**
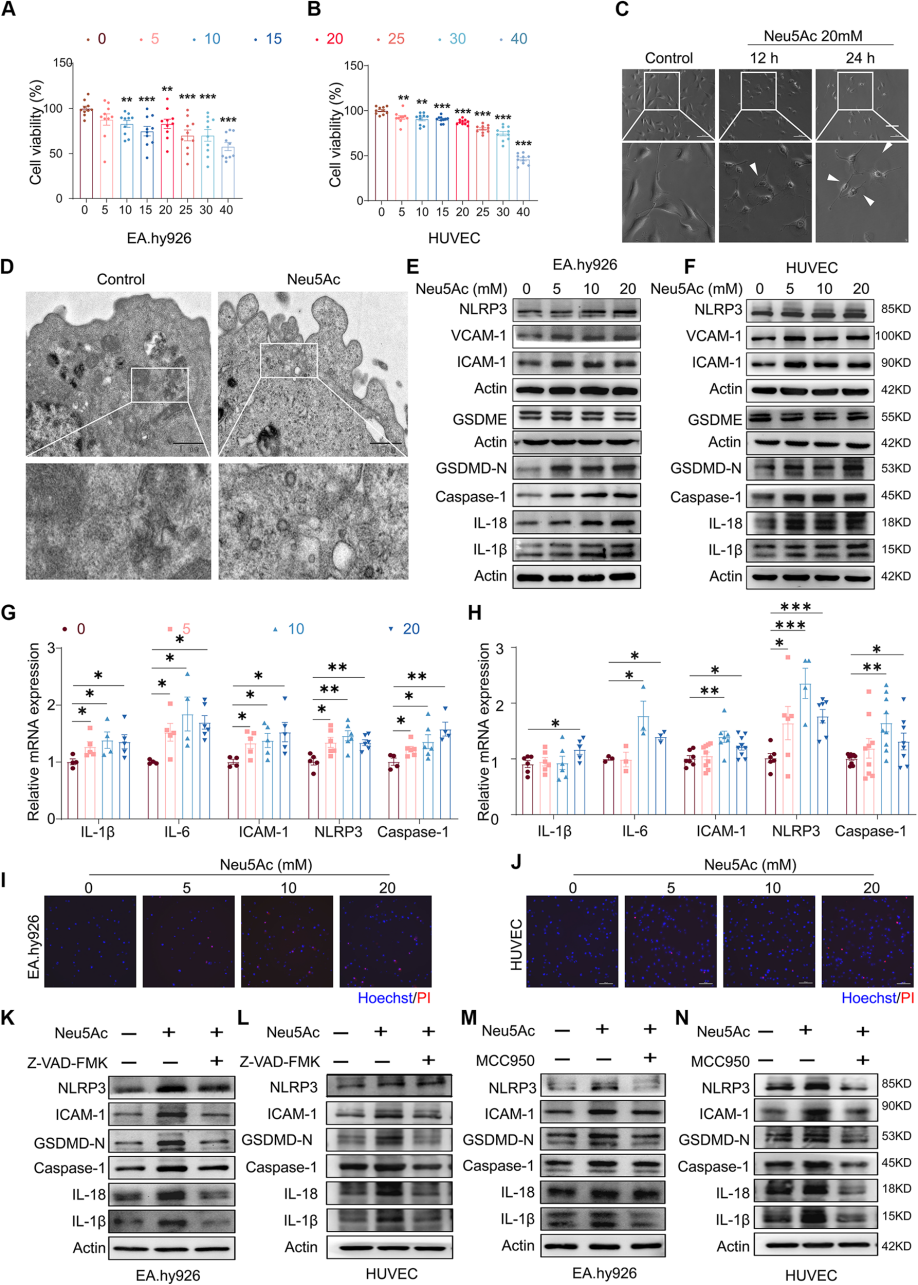
Neu5Ac contributed to endothelial inflammatory injury through NLRP3 dependent pyroptosis pathway. **A**, **B** ECs were treated with the indicated concentrations (0, 5, 10, 15, 20, 25, 30 and 40 mM) of Neu5Ac for 12 h. The cytotoxicity was measured by using a CCK-8 detection kit. **C** The morphology of EA.hy926 cells treated with 20 mM Neu5Ac in vitro for 12 or 24 h under optical microscopy. Bar = 100 µm. **D** The morphology of EA.hy926 cells treated with 20 mM Neu5Ac for 12 h under transmission electron microscopy. Bar = 1 µm. **E**, **F** Western blot analysis of pyroptosis markers (GSDMD-N, GSDME, NLRP3 inflammasome, Caspase-1, IL-1β, and IL-18) and pro-inflammatory adhesion molecules (ICAM-1 and VCAM-1) protein expression in ECs treated with indicated concentrations of Neu5Ac for 12 h. **G**, **H** qRT-PCR analysis of IL-1β, IL-6, Caspase-1, NLRP3 and ICAM-1 expression in ECs treated with indicated concentrations of Neu5Ac for 12 h. **I**, **J** Pore formation in the cell membrane of HUVECs treated with indicated concentrations of Neu5Ac for 12 h was observed by Hoechst/PI staining. Bar = 100 μm. **K**, **L** ECs were preincubated with or without Z-VAD-FMK (10 µM) for 1 h before Neu5Ac (20 mM) treatment for 12 h, IL-1β, IL-18, Caspase-1, NLRP3, GSDMD-N and ICAM-1 protein expression were assessed in the ECs by western blot. **M**, **N** ECs were preincubated with or without MCC950 (10 µM) for 1 h before Neu5Ac (20 mM) treatment for 12 h, IL-1β, IL-18, Caspase-1, NLRP3, GSDMD-N and ICAM-1 protein expression were assessed in the ECs by western blot. Data were analyzed using unpaired two-tailed Student’s *t*-tests or one-way ANOVA tests, and presented as the means ± SEM. *p < 0.05 was considered significant, **p < 0.01, ***p < 0.001.

To investigate whether pyroptosis activation involved in endothelial injury following Neu5Ac treatment, we initially verified the effect of Neu5Ac on viability of vascular endothelial cells. As shown in Fig. 1A, B, Neu5Ac (5–20 mM) displayed significantly cytotoxicity on EA.hy926 and human umbilical vein endothelial cells (HUVECs) in a dose-dependent manner, which confirmed in our previous work [14, 15]. Meanwhile, we found that HUVECs exhibited balloon-like protrusions on cell membrane and experienced the leakage of cytosolic contents following Neu5Ac treatment as shown in Fig. 1C, D. This observation strongly indicated the initiation of a specific type of programmed cell death, known as pyroptosis [28]. Therefore, we further examined the expression of pyroptosis markers (GSDMD-N, GSDME, NLRP3 inflammasome, Caspase-1, IL-1β, and IL-18) and pro-inflammatory adhesion molecules (ICAM-1 and VCAM-1) after Neu5Ac treatment. Results from western blotting showed that Neu5Ac increased the expression of above pyroptosis markers and ICAM-1, VCAM-1 as well. And the expression of GSDME remained relatively stable and showed minimal changes (Figs. 1E, F and S1A–D). In addition, results from monocyte–endothelial adhesion analysis indicated that Neu5Ac increased the ability of ECs adhesion to monocyte (Fig. S1E–H). qRT-PCR analysis also confirmed the above observations that Neu5Ac increased the mRNA levels of IL-1β, IL-6, Caspase-1, NLRP3 and ICAM-1 as shown Fig. 1G, H. Moreover, Hoechst/PI staining showed that Neu5Ac increased the number of PI-positive ECs (Figs. 1I, J and S1I, J). Together, these results indicated that Neu5Ac might induce pyroptosis activation and then contributes to endothelial inflammatory injury.

Next, we utilized pyroptosis inhibitor, Z-VAD-FMK, to further verify the involvement of pyroptosis in inducing endothelial inflammatory injury. The results from Fig. S1K–M showed that Z-VAD-FMK effectively reversed the number of pyroptotic cells and inhibited the expressions of GSDMD-N, Caspase-1, IL-1β, IL-18 and ICAM-1 in ECs following Neu5Ac treatment (Figs. 1K, L and S1N, O). In addition, the number of monocyte adhesion to endothelial were also decreased (Fig. S1P–R). MCC950, a small molecule inhibitor of NLRP3 [29], was found to effectively reverse the presence of PI-positive pyroptotic ECs, as illustrated in Fig. S2A–C. Additionally, MCC950 led to a reduction of Caspase-1, GSDMD-N, IL-1β, IL-18 and ICAM-1, as depicted in Fig. 1M, N, and in Fig. S2D, E. Furthermore, this reduction in pyroptotic markers correlated with a decrease in monocyte–endothelial adhesion ability, as shown in Fig. S2F–H. Together, these results strongly suggested that endothelial pyroptosis induced by Neu5Ac was regulated through NLRP3/Caspase-1/GSDMD signaling pathway.

As acknowledged, the abnormal sialic acid metabolism has affected on endogenous Neu5Ac levels. UDP-GlcNAc 2-epimerase (GNE) and CMP-Neu5Ac synthase (CMAS) are pivotal enzymes in the biosynthesis of Neu5Ac and Neu5Ac-conjugated glycans respectively [30]. In our present study, we found that CMAS siRNA or overexpression of GNE in ECs could increase intracellular Neu5Ac levels (Fig. S2I–K). Interestingly, the increasing of intracellular Neu5Ac also promoted the pyroptosis activation in ECs, as evidenced by Fig. S2L, M. Together, these results suggested that pyroptosis activation was highly associated with elevated Neu5Ac levels in ECs.

### Neu5Ac promoted atherosclerosis development through pyroptosis activation in ApoE^−/−^ mice

**Fig. 2:**
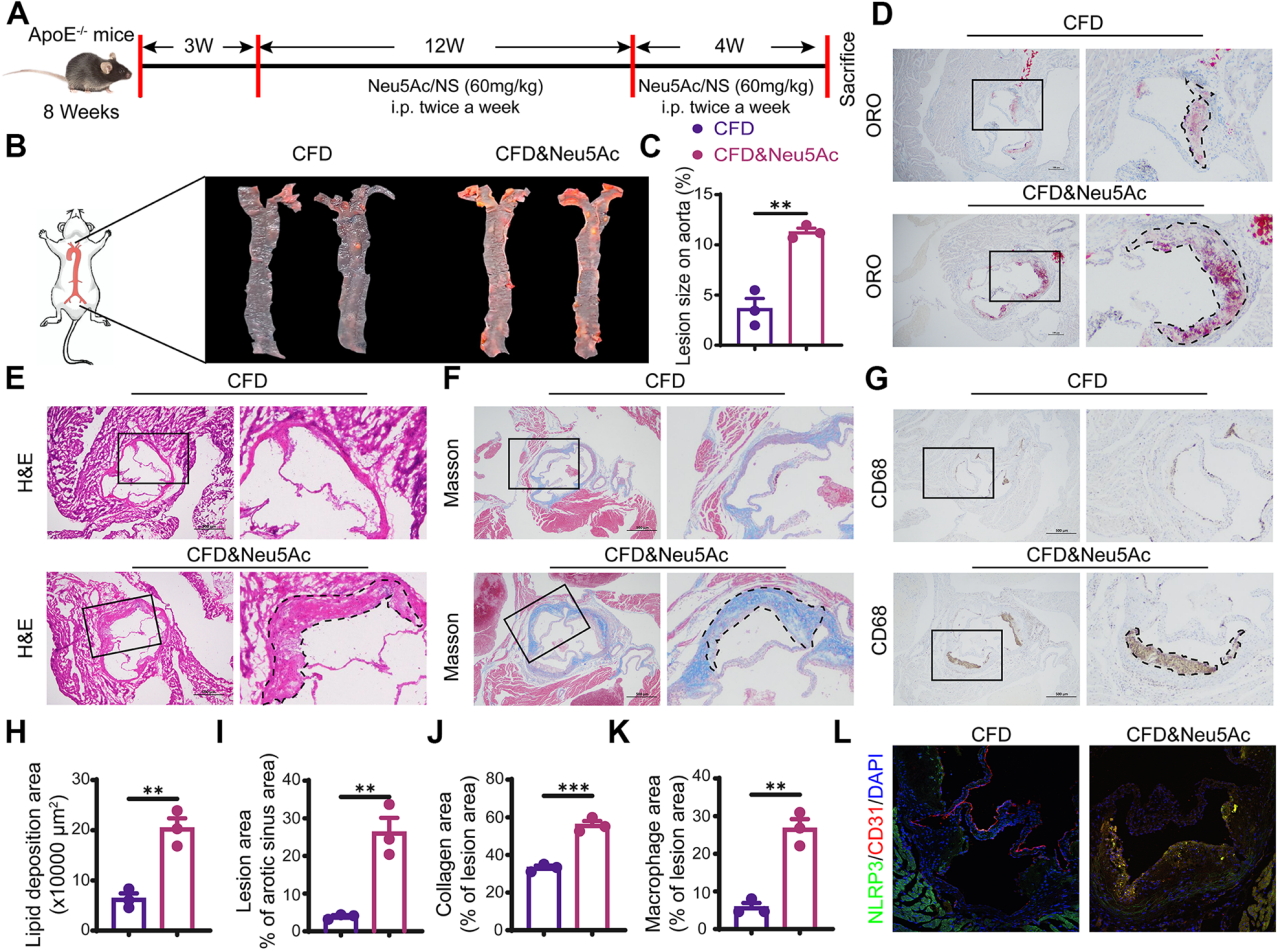
Neu5Ac activated endothelial pyroptosis and promoted AS development in ApoE^−/−^ mice. **A** Diagram showing generation of Neu5Ac-treated ApoE^−/−^ mice. Oil Red O staining atherosclerotic and quantitative analysis of lesions of aorta en face (**B**, **C**) and aortic root (**D**, **H**) in ApoE^−/−^ mice injected with 0.9% N.S. (*n* = 3) or Neu5Ac (*n* = 3); HE staining (**E**, **I**), Masson staining (**F**, **J**) and CD68 staining (**G**, **K**) and quantitative analysis of atherosclerotic lesions of aortic roots in ApoE^−/−^ mice injected with 0.9% N.S. (*n* = 3) or Neu5Ac (*n* = 3), with quantitative data at below. Bar = 100 μm. **L** NLRP3 co-immunoflurorescence staining with CD31 of aortic root from ApoE^−/−^ mice injection with 0.9% N.S. (*n* = 3) or Neu5Ac (*n* = 3). Bar = 100 µm. Data were analyzed using unpaired two-tailed Student’s *t*-tests or one-way ANOVA tests, and presented as the means ± SEM. **p  <  0.01, ***p  <  0.001 were considered significant.

In line with previous studies [12, 13], we also observed a significantly increasing of Neu5Ac level in hyperlipidemic patients or hyperlipidemia mice models compared with health donors or normal mice, individually (Fig. S3A, B). To further investigate the effect of Neu5Ac on endothelial pyroptosis in vivo, we established a mouse model characterized by elevated Neu5Ac levels in circulation, as previously described (Figs. 2A and S3C). H&E staining showed that Neu5Ac injection could induce “vacuolar” changes in liver cells and lightly vacuolar-like degradation appearance in kidney, indicating that Neu5Ac induced lipids accumulation in livers (Fig. S3D–F). Blood biochemical analyses revealed an increasing level of triglyceride and a decreasing trend of alanine aminotransferase (ALT) in ApoE^−^^/−^ mice following Neu5Ac injection (Fig. S3G), while the levels of total cholesterol, HDL cholesterol, LDL cholesterol, and glucose remained lightly changed (Fig. S3G, H). These findings suggested that Neu5Ac treatment lead to dysregulation of blood lipids and liver injury. In addition, we also observed significant lipid accumulation within aortic tree and aortic sinus (Fig. 2B–D, H) in Neu5Ac-treated ApoE^−^^/−^ mice. This was accompanied by increased plaque lesion size, heightened collagen and greater infiltration of macrophage in the aortic roots (Fig. 2E–G, I–K). These observations also strongly implied that Neu5Ac could enhance the formation of atherosclerotic plaques as we confirmed before [14, 15]. Concurrently, increased TUNEL-positive cells (Fig. S3I) and increased expression NLRP3 (Figs. 2L and S4A–D) were observed in the aortic roots of ApoE^−/−^ mice with Neu5Ac injection, indicating that Neu5Ac also induced pyroptosis activation in vivo. Taken together, these findings confirmed that Neu5Ac activated pyroptotic pathway and then promoted ECs damage through NLRP3/Caspase-1/GSDMD pathway both in vivo and in vitro, thereby contribute to AS development.

### Neu5Ac induced endothelial mitochondrial damage and contributed to pyroptosis activation

**Fig. 3:**
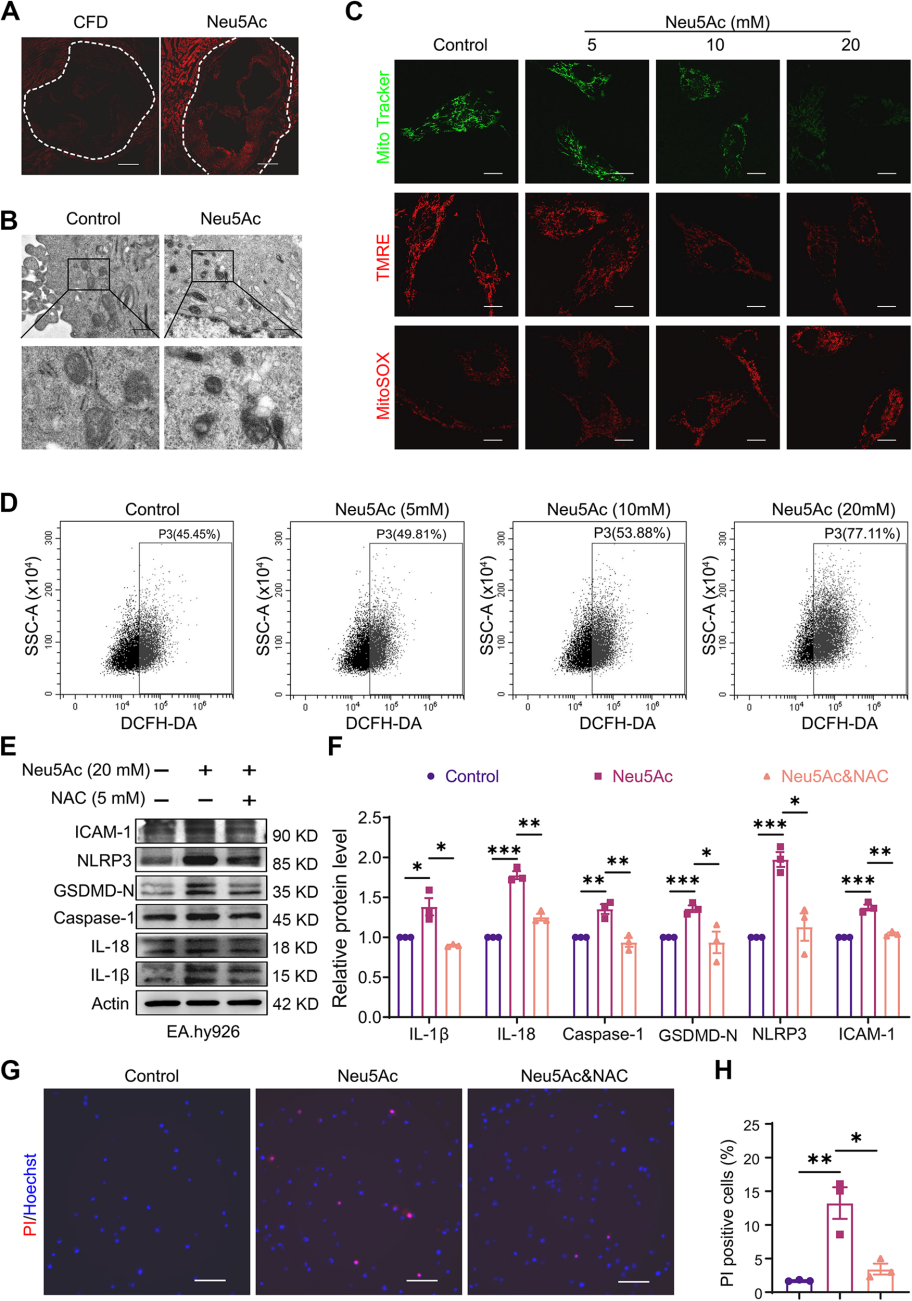
Neu5Ac induced endothelial mitochondrial damage and contributed to pyroptosis activation. **A** DHE staining showed intracellular superoxide production in aortic root from 0.9% N.S. (*n* = 3) or Neu5Ac-injected ApoE^−/−^ mice (*n* = 3). Bar = 500 µm. **B** Electron microscopy of the mitochondria of Neu5Ac-treated EA.hy926 cells. Bar = 1 µm. **C** EA.hy926 cells were treated with indicated concentrations of Neu5Ac for 12 h, mitochondrial mass was labeled by MitoTracker Green; the mitochondrial membrane potential was assayed by TMRE; the mitochondrial ROS level was detected by mitoSOX. Bar = 31.75 µm. **D** The intracellular ROS were measured by DCFH-DA probe in Neu5Ac-treated EA.hy926 cells. EA.hy926 cells were preincubated with or without ROS scavenger NAC (5 mM) for 1 h before Neu5Ac (20 mM) treatment for 12 h, IL-1β, IL-18, Caspase-1, NLRP3, GSDMD-N and ICAM-1 protein expression were assessed by western blot, with quantitative data at right (**E**, **F**); and cell death in HUVEC was detected by PI staining, with quantitative data at below (**G**, **H**). Bar = 100 µm. Data were analyzed using unpaired two-tailed Student’s *t*-tests or one-way ANOVA tests, and presented as the means ± SEM. *p < 0.05 was considered significant, **p < 0.01, ***p < 0.001.

Mitochondria are recognized as the main source of cellular ROS and serve as vital bioenergetic organelles for redox homeostasis [31]. Once mitochondria damaged, the increased ROS could lead to the activation of NLRP3 inflammasome and further promoted the initiation of pyroptosis [32]. In the present study, we utilized dihydroethidium (DHE) staining and found an augmentation of ROS in aortic roots from mice with Neu5Ac injection compared to control mice (Figs. 3A and S5B). We further investigated the impact of Neu5Ac on endothelial mitochondrial morphology and function in vitro. Transmission electron microscopy unveiled the absence of both membrane and inner carinulae of mitochondria in Neu5Ac-treated ECs (Fig. 3B). Intracellular ROS and mitochondrial ROS (mtROS) were both elevated in Neu5Ac-treated ECs, concomitant with a reduction of mitochondrial membrane potential and mitochondrial mass (Figs. 3C, D and S5A). These results from in vivo and in vitro strongly indicated that Neu5Ac induced mitochondrial dysfunction, and promoted ROS production.

To further validate the role of ROS in NLRP3 activation and pyroptosis induced by Neu5Ac, we utilized a ROS scavenger, *N*-acetylcysteine (NAC). As shown in Figs. 3E, F and S5H, I, NAC pretreatment notably downregulated the expression of NLRP3, Caspase-1, IL-1β, IL-18 and ICAM-1 and led to a significant reduction of PI-positive cells’ proportion, and improved mitochondrial function as well (Figs. 3G, H and S5D–F). Additionally, the number of monocyte adhesion to endothelial cells was also decreased upon NAC pretreatment (Fig. S5E, G, H). These findings collectively indicated that Neu5Ac induced endothelial cell pyroptosis through mitochondrial damage and subsequent ROS generation.

### GLS2-mediated glutaminolysis facilitated pyroptosis by promoting mtROS formation

**Fig. 4:**
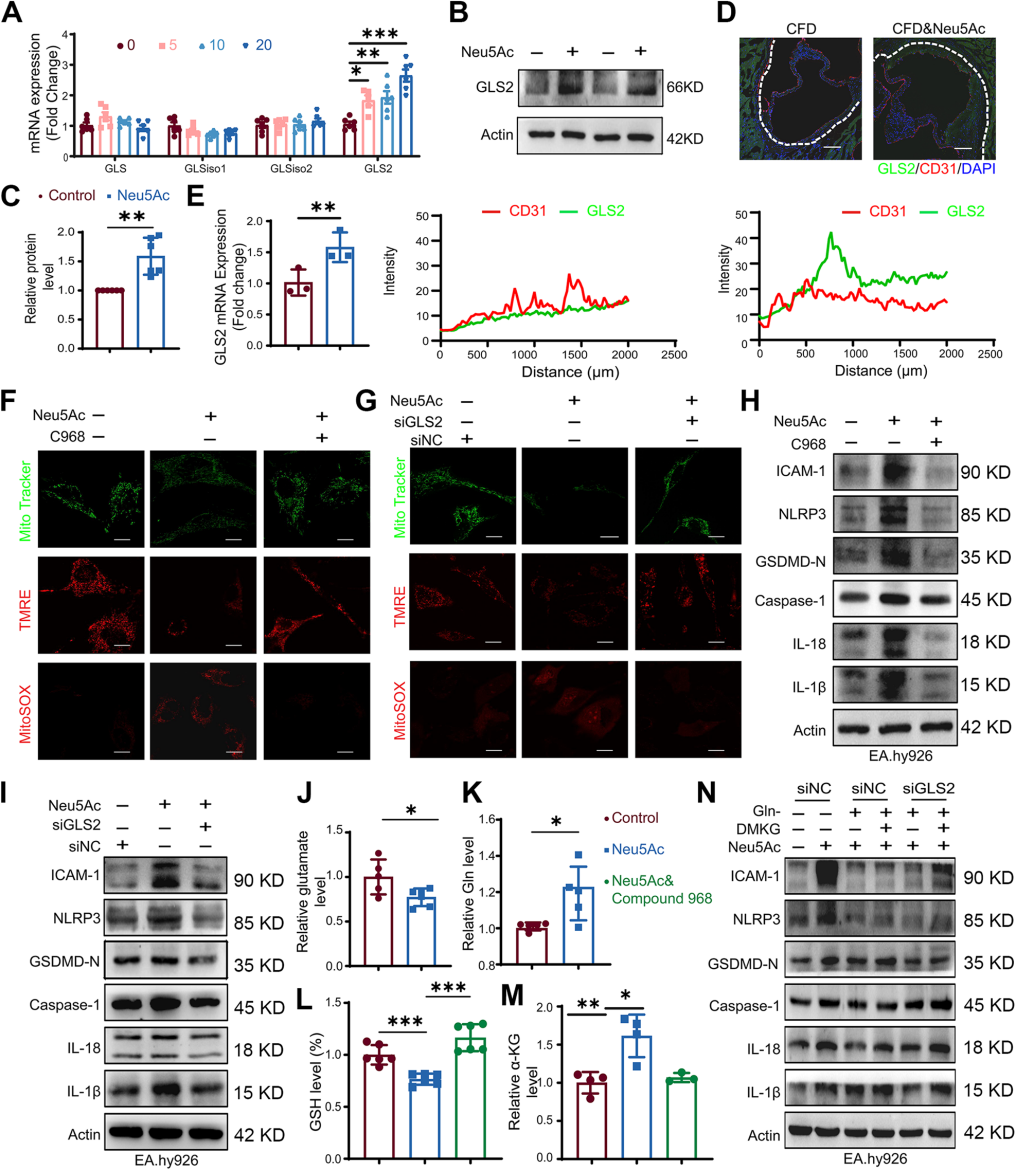
GLS2-mediated glutaminolysis facilitated pyroptosis by increasing mtROS levels. **A** qRT-PCR analysis of key enzymes of glutaminolysis process (GLS, GLSiso1, GLSiso2 and GLS2) in EA.hy926 cells treated with indicated concentrations of Neu5Ac (0, 5, 10 and 20 mM) for 12 h. **B**, **C** EA.hy926 cells were treated with Neu5Ac (20 mM) for 12 h, GLS2 expression was assessed by western blot, with quantitative data at below. **D** CD31 and GLS2 immunostaining of aortic root from 0.9% N.S. (*n* = 3) or Neu5Ac-injected ApoE^−^^/−^ mice (*n* = 3), with quantitative data at below. Bar = 100 µm. **E** qRT-PCR analysis of GLS2 in aortic artery from 0.9% N.S. (*n* = 3) or Neu5Ac-injected ApoE^−^^/−^ mice (*n* = 3). EA.hy926 cells were preincubated with or without Compound 968 (10 µM) for 1 h or transfected with GLS2 siRNA before Neu5Ac (20 mM) treatment for 12 h, mitochondrial mass, membrane potential and mitochondrial ROS production were labeled, bar = 31.75 µm (**F**, **G**); IL-1β, IL-18, Caspase-1, NLRP3, GSDMD-N and ICAM-1 protein expression were assessed by western blot (**H**, **I**). EA.hy926 cells were preincubated with or without Compound 968 (10 µM) for 1 h before Neu5Ac (20 mM) treatment for 12 h, the intracellular level of glutamate (**J**) and glutamine (**K**) were assessed by ELISA; the intracellular level of GSH was measured by using Glutathione Assay Kit (**L**); the intracellular level of α-KG was measured by LC/MS (**M**). **N** Western blot analysis of IL-1β, IL-18, Caspase-1, NLRP3, GSDMD and ICAM-1 of EA.hy926 cells deprived of Gln (-Gln) or transfected with GLS2 siRNA followed by Neu5Ac (20 mM) treatment for 12 h in the presence or absence of 4 mM DMKG for 3 h. Data were analyzed using unpaired two-tailed Student’s *t*-tests or one-way ANOVA tests, and presented as the means ± SEM. *p < 0.05 was considered significant, **p < 0.01, ***p < 0.001.

Glutamine metabolism is found to be critical for cellular ROS homeostasis as well as mitochondrial function [32]. To further elucidate the molecular mechanisms of Neu5Ac induced pyroptosis, we assessed the effects of Neu5Ac on the expression of key enzymes (GLS, GLSiso1, GLSiso2, GLS2) involving in glutaminolysis pathway [33]. qRT-PCR analysis revealed that the mRNA levels of GLS2 but not GLS was increased in Neu5Ac-treated ECs (Fig. 4A). Western blotting and immunofluorescence staining also confirmed the increasing of GLS2 levels in ECs after Neu5Ac treatment (Figs. 4B, C and S6A). In line with in vitro findings, in vivo experiments also demonstrated the increasing expression of GLS2 in aortic roots and aortic artery from ApoE^−/−^ mice following Neu5Ac treatment (Fig. 4D, E). Given the evidence that GLS2 is mainly located in mitochondria [34], we hypothesized that GLS2 might be important in Neu5Ac-induced pathological processes. Therefore, we employed a pharmaceutical inhibitor of GLS2, Compound 968 and GLS2 siRNA as well (Fig. S6H) in ECs. Our results showed that both GLS2 siRNA and Compound 968 could ameliorate mitochondrial dysfunction and reduce ROS production, inflammation and pyroptosis activation induced by Neu5Ac (Figs. 4F–I and S6B–G, S7A–C). These findings further indicated that GLS2-mediated glutaminolysis pathway was highly involved in mitochondrial dysfunction induced by Neu5Ac.

To further elucidate the mechanism by which GLS2 regulated mtROS production, we detected the levels of several glutaminolysis-related metabolites. Compared to the control group, the intracellular level of glutamate was decreased (Fig. 4J), while the glutamine level was increased in ECs after Neu5Ac treatment (Fig. 4K). These results suggested that excess glutamate utilization may occur in endothelial pyroptosis activation induced by Neu5Ac. Therefore, we hypothesize that GLS2 may contribute to mitochondrial oxidation and/or reduce abolishment of oxidative stress, and then promote mtROS production. In contrast to the previous report indicating that GLS2 exerts antioxidant activity via GSH [35], we observed a decrease in GSH levels in Neu5Ac-treated ECs, which was rescued by GLS2 inhibition (Fig. 4L). The above results implied that reduced GSH levels might contribute to mtROS accumulation. Concurrently, in line with the enhanced mRNA levels of such aminotransferases like glutamic-oxaloacetic transaminase 2 (GOT2), glutamate pyruvate transaminase 2 (GPT2) and glutamate dehydrogenase (GLUD1), we also observed an increase of α-KG levels in Neu5Ac-treated ECs, and were reversed by GLS2 inhibition (Figs. 4M and S7D–F). These results suggested that more glutamate may be diverted into the tricarboxylic acid (TCA) cycle. Together, the present results demonstrated the crucial role of GLS2 in accelerating mitochondrial damage and pyroptosis induced by Neu5Ac.

α-KG is reported to be able to trigger pyroptosis [36]. Thus, whether GLS2 affected pyroptosis by modulating α-KG production is still not clear. Therefore, we investigated whether Neu5Ac-induced pyroptosis could be exacerbated by α-KG. We found that α-KG supplementation, using analog DM-KG, effectively rescue pyroptosis in Gln-deprived and GLS2 knockdown ECs under Neu5Ac exposure (Figs. 4N and S7G). Based on these findings, we proposed that Neu5Ac induced mtROS-dependent pyroptosis by regulating GLS2 expression, leading to a-KG production and restraining GSH levels.

### GLS2 knockdown ameliorated atherosclerosis progression in ApoE^−/−^ mice

**Fig. 5:**
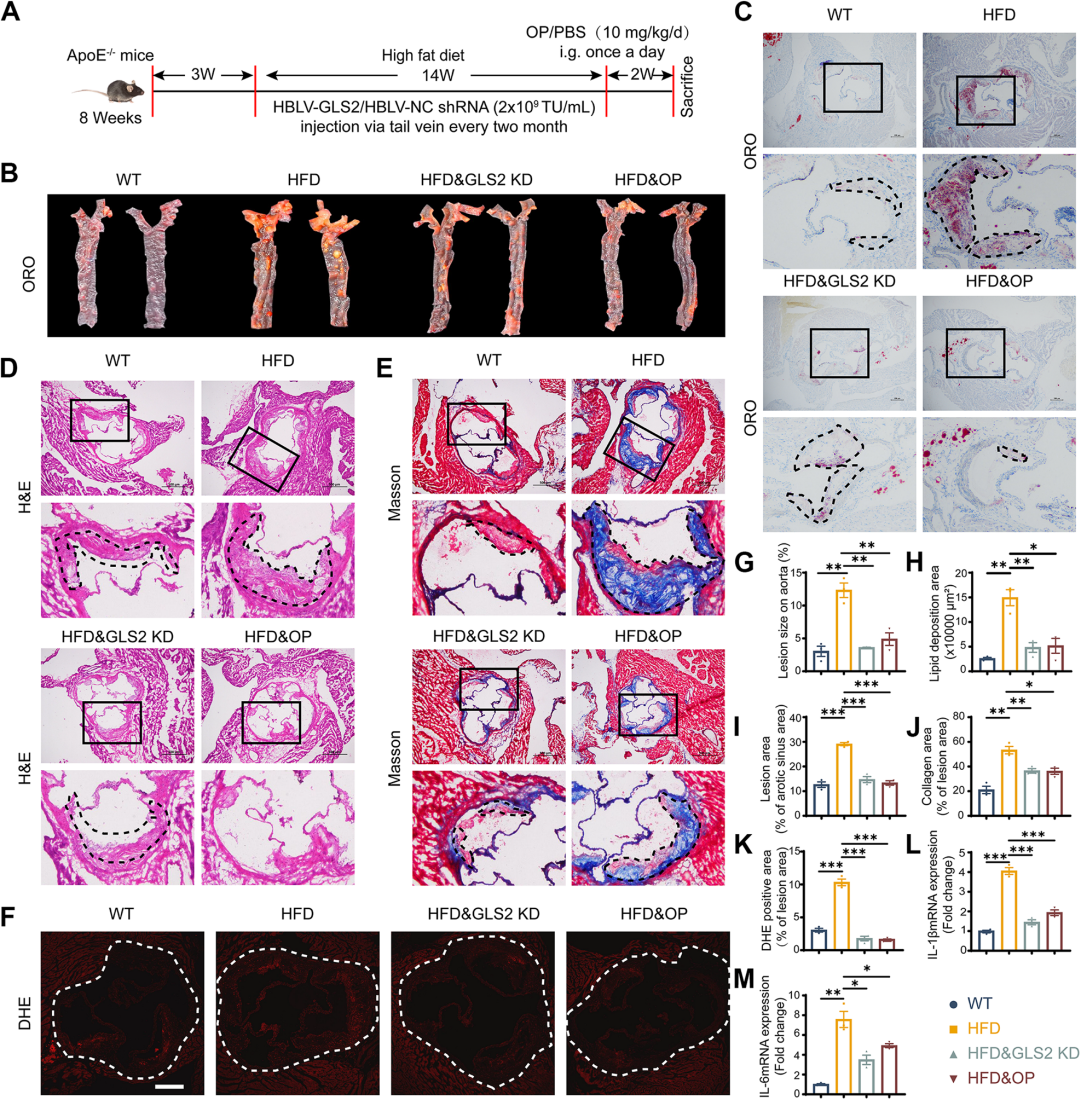
GLS2 knockdown ameliorated AS progression in ApoE^−^^/−^ mice. **A** Diagram showing generation and pathologic analysis of HFD, GLS2 KD, OP-administrated ApoE^−/−^ mice (*n* ≥ 3). Oil Red O staining and quantitative data of atherosclerotic lesions of aorta en face (**B**, **G**) and aortic root (**C**, **H**) in WT, HFD, GLS2 KD and OP-administrated ApoE^−/−^ mice. Bar = 100 µm. HE staining (**D**, **I**), Masson staining (**E**, **J**) and DHE staining (**F**, **K**) of atherosclerotic lesions of aortic root, with quantitative data right. **L**, **M** qRT-PCR analysis of IL-1β and IL-6 expression in aortic artery from WT, HFD, GLS2 KD and OP-administrated ApoE^−/−^ mice. Data were analyzed using unpaired two-tailed Student’s *t*-tests or one-way ANOVA tests, and presented as the means ± SEM. *p < 0.05 was considered significant, **p < 0.01, ***p < 0.001.

To further explore the role of GLS2 in AS development, we utilized a loss-of-function approach by using lentivirus encoding GLS2 short hairpin RNA (shRNA) (HBLV-GLS2 shRNA), with HBLV-Control shRNA serving as a negative control (Fig. 5A). Immunofluorescence results confirmed that HBLV-GLS2 shRNA was successfully transduced into ECs (Fig. S8A). The mRNA level of GLS2 was also observed to decreased in aortic artery from GLS2 KD ApoE^−/−^ mice (Fig. S8B). Subsequently, we examined the impact of GLS2 knockdown on atherosclerotic phenotypes in ApoE^−/−^ mice fed on high-fat diet. No obvious body weight difference or liver and kidney injury were observed (Fig. S8C–H). Notably, GLS2 knockdown could alleviate the lipid accumulation (Fig. S8G) in liver cells and led to a decrease of serum lipids levels including triglycerides, total cholesterol, LDL cholesterol and glucose in mice feeding with HFD, while the level of HDL cholesterol randomly changed (Fig. 5H–M).

Furthermore, GLS2 KD ApoE^−^^/−^ mice exhibited reduced plaque areas in aortic and aortic roots, as well as decreased superoxide radical production in aortic roots, along with a reduction of collagen content in aortic roots compared to control mice (Fig. 5B–K). TUNEL staining further confirmed a decrease of TUNEL-positive cells in the aortic roots from GLS2 KD ApoE^−/−^ mice (Fig. S8N). In addition to these improvements, we also observed the expression of IL-1β and IL-6 were significantly reduced in aortic artery of GLS2 KD ApoE^−^^/−^ mice (Fig. 5L, M). Pyroptosis markers (NLRP3, Caspase-1 and IL-18) and adhesion molecules (ICAM-1) were also markedly decreased in the aortic roots of GLS2 KD ApoE^−/−^ mice (Fig. S9A–H). Taken together, these findings suggested that GLS2 was highly involved in AS progression with Neu5Ac accumulation and, knockdown of GLS2 could ameliorate lipid accumulation and AS progression.

### Neu5Ac promotes GLS2 activation via SIRT3/FOXO3a pathway

**Fig. 6:**
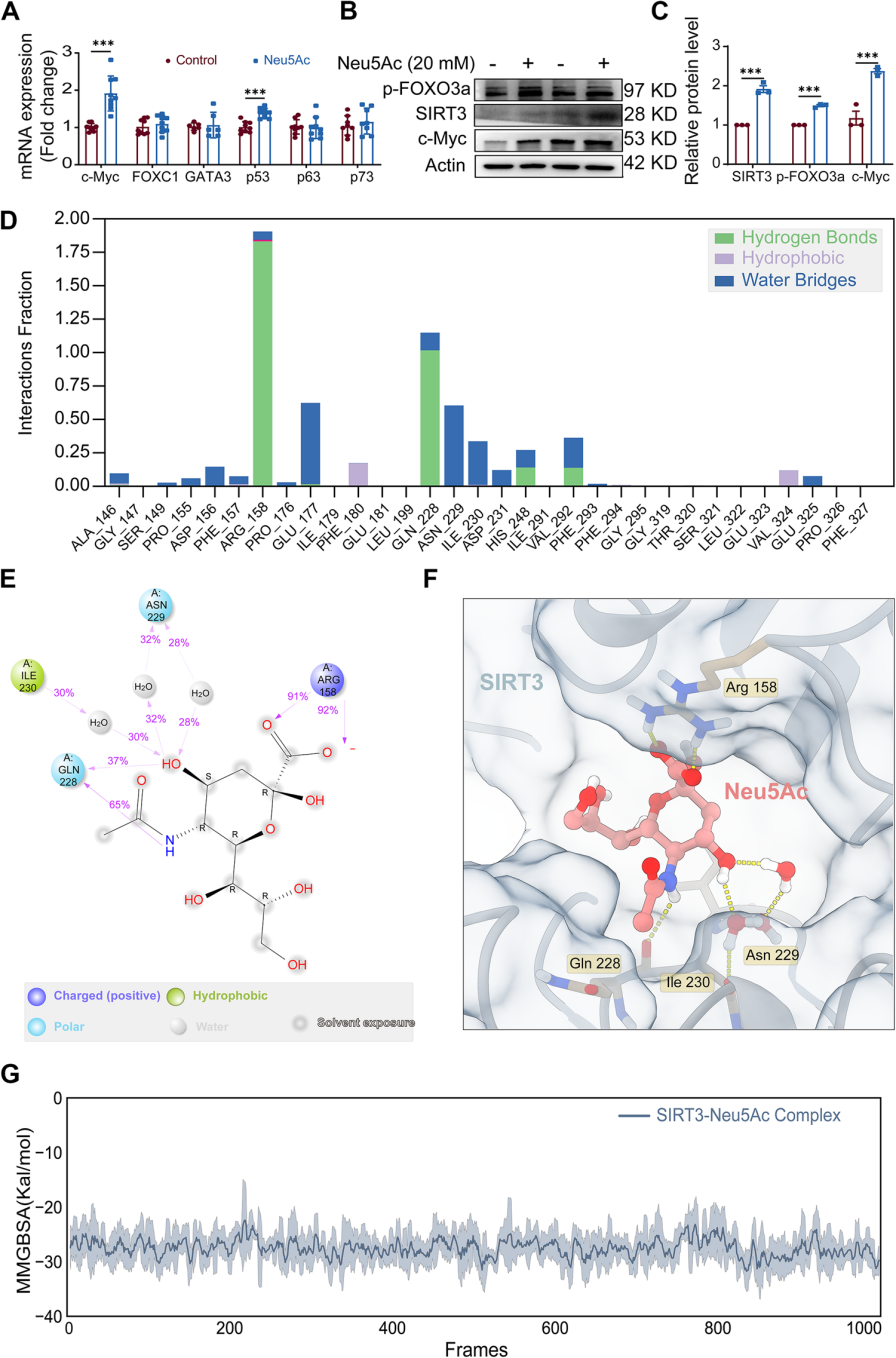
Neu5Ac bind to SIRT3 and activated following pathway. **A** qRT-PCR analysis of transcription factors of GLS2 in EA.hy926 cells treated with Neu5Ac (20 mM) for 12 h. **B**, **C** EA.hy926 cells were treated with or without Neu5Ac (20 mM), SIRT3, p-FOXO3a, c-Myc and GLS2 protein expression were assessed by western blot, with quantitative data at right. The molecular docking techniques were utilized to investigate the potential interaction between Neu5Ac and SIRT3, the amino acids that play an important role in the binding of Neu5Ac in the stable interval of molecular dynamics locus were analyzed (**D**–**F**); the 1000-frame locus of the last 10 ns of molecular dynamics of small molecule Neu5Ac and protein SIRT3 was extracted, and the free binding energy of Neu5Ac and SIRT3 was calculated (**G**). Data were analyzed using unpaired two-tailed Student’s *t*-tests or one-way ANOVA tests, and presented as the means ± SEM. *p < 0.05 was considered significant, **p < 0.01, ***p < 0.001.

Given the critical role of GLS2 in AS progression induced by Neu5Ac, we further elucidated the mechanisms underlying Neu5Ac regulates GLS2 expression. The results showed that the protein stability of GLS2 barely changed upon Neu5Ac treatment (Fig. S10A). However, we observed a significantly increase in GLS2 mRNA levels (Fig. 4A), suggesting that GLS2 might be transcriptionally regulated by Neu5Ac. Therefore, based on the literature reports before, we examined several transcription candidates for regulating GLS2 levels [37–41]. As shown in Fig. 6A, levels of transcription factors like p63, p73, FOXC1, GATA3 did not changed after Neu5Ac treatment, while the level of c-Myc and p53 exhibited a significant increasing. Notably, FOXO3a has been reported to counteract Myc in promoter region and suppressed the expressions of mitochondrial genes through regulating SIRT3 pathway [42, 43]. Therefore, we hypothesized that Neu5Ac might regulate GLS2 expression through SIRT3/FOXO3a/c-Myc pathway. Then, we utilized western blots analysis to confirm our hypothesis. As shown in Fig. 6B, C, the expression of SIRT3, c-Myc and p-FOXO3a was increased in ECs after Neu5Ac treatment. Next, the molecular docking techniques were utilized to investigate the potential interaction between Neu5Ac and SIRT3. The results showed that Neu5Ac could bind to SITR3 and the structure of SIRT3–Neu5Ac complex appeared to be relatively stable (Fig. S11A, B). Besides, Neu5Ac was mainly bound to the less flexible region of SIRT3, and the more flexible region mainly located at the loop region outside the active site, indicating that Neu5Ac might stabilize the protein structure of SIRT3 (Fig. S11C, D). Further analysis revealed that specific amino acids, including Arg158, Glu177, Gln228, Asn229 and Ile230, played important roles in stabling Neu5Ac/SIRT3 complex (Fig. 6D–F). In addition, the binding free energy of Neu5Ac and SIRT3 was about −30 kcal/mol, further illustrated the binding was stable (Fig. 6G).

**Fig. 7:**
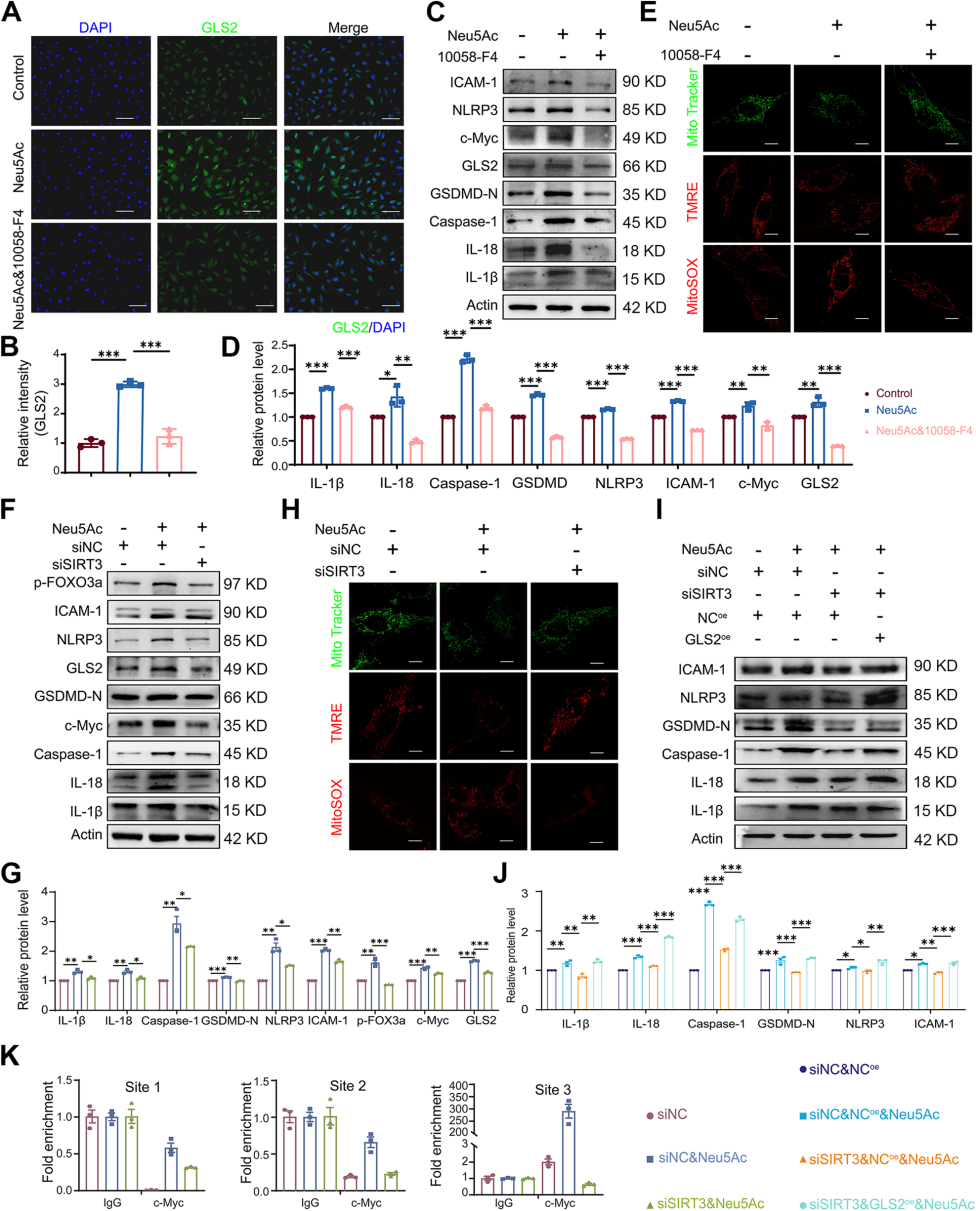
Neu5Ac promoted GLS2 activation via SIRT3/FOXO3a/c-Myc pathway. EA.hy926 cells were preincubated with or without 10058-F4 (60 µM) for 1 h before Neu5Ac (20 mM) treatment for 12 h, GLS2 and DAPI immunostaining were performed, with quantitative data at below; bar = 100 µm (**A**, **B**); IL-1β, IL-18, Caspase-1, NLRP3, GSDMD-N, GLS2 and ICAM-1 protein expression were assessed by western blot, with quantitative data at below (**C**, **D**); mitochondrial mass, membrane potential and mitochondrial ROS production were labeled by MitoTracker Green, TMRE and mitoSOX respectively; bar = 31.75 µm (**E**). EA.hy926 cells were transfected with SIRT3 siRNA or NC siRNA before Neu5Ac (20 mM) treatment for 12 h, IL-1β, IL-18, Caspase-1, NLRP3, GSDMD-N, GLS2, p-FOXO3a and ICAM-1 protein expression were assessed by western blot and quantified (**F**, **G**). Mitochondrial mass, membrane potential and mitochondrial ROS production were labeled by MitoTracker Green, TMRE and mitoSOX respectively; bar = 31.75 µm (**H**). **I**, **J** Western blot analysis of IL-1β, IL-18, Caspase-1, NLRP3, GSDMD-N and ICAM-1 of EA.hy926 cells transfected with GLS2 plasmid and SIRT3 siRNA followed by Neu5Ac (20 mM) treatment for 12 h. **K** EA.hy926 cells were transfected with SIRT3 siRNA or NC siRNA before Neu5Ac (20 mM) treatment for 12 h, Chromatin immunoprecipitation followed by quantitative PCR (ChIP-qPCR) was performed with an antibody for c-Myc and antibody for IgG as the negative control. Data were analyzed using unpaired two-tailed Student’s *t*-tests or one-way ANOVA tests, and presented as the means ± SEM. *p < 0.05 was considered significant, **p < 0.01, ***p < 0.001.

As acknowledged, phosphorylation of FoxO3a could increase nuclear export and reduce transcriptional activity [44]. To further elucidate the role of c-Myc, we employed 10058-F4, a c-Myc inhibitor, for further experiments. We found that 10058-F4 effectively inhibited the expression of GLS2 and alleviated the expression of makers associated with mitochondrial injury, pyroptosis and inflammation (Figs. 7A–D, H and S10B, C). Furthermore, to better validate the activation of SIRT3/FOXO3a pathway in endothelial injury induced by Neu5Ac, we applied SIRT3 siRNA (Fig. S10D) and SIRT3 inhibitor. The result indicated that both SIRT3 siRNA and pharmacological inhibition of SIRT3 could significantly inhibited the expressions of p-FOXO3A, c-Myc and GLS2 in Neu5Ac-treated ECs, and subsequently reversed mitochondrial damage, pyroptosis and inflammation levels induced by Neu5Ac (Figs. 7E, F, H and S10E,S12A–F). Importantly, GLS2 overexpression could inhibit the protective effect of SIRT3 suppression (Figs. 7I, J and S10F). Chromatin immunoprecipitation assay (ChIP assay) further confirmed that Neu5Ac promoted the binding of c-Myc to GLS2 promoter region, and SIRT3 knockdown reversed the above binding activity (Fig. 7K), suggesting that c-Myc should be the potential transcription factor of GLS2 and could be regulated by SIRT3 in Neu5Ac-treated ECs. Interestingly, cells with CMAS siRNA or GNE overexpression could also promote the expression of p-FOXO3a, c-Myc and GLS2 (Fig. S10G). Taken together, these findings provide evidence that Neu5Ac could bind to SIRT3 and then activated FOXO3a/c-Myc pathway, ultimately promote GLS2-associated glutaminolysis disorder.

### Pharmacological inhibition of Neu5Ac is a promising auxiliary medication for AS treatment

Neuraminidases (NEUs) are the family of enzymes responsible for cleaving sialic acid from cell surfaces [45, 46]. In the present study, we observed the increased expressions of NEUs like NEU3 in ApoE^−/−^ mice feeding with HFD or with Neu5Ac injection (Fig. S8I, J). Thus, we deployed oseltamivir phosphate (OP), widely used for anti-influenza and also known as NEUs inhibitor [45]. Of note, OP treatment reduced Neu5Ac production (Fig. S8K) and effectively alleviated atherosclerotic lesions in ApoE^−^^/−^ mice feeding with HFD (Fig. 5B, C, G, H). Additionally, OP treatment led to the decrease of TUNEL-positive cells, collagen area and production of superoxide radicals in aortic roots of ApoE^−/−^ mice with HFD feeding (Figs. 5D–F, I–K and S8N). Moreover, mRNA levels of IL-1β and IL-6 were both decreased in aortic arteries of ApoE^−/−^ mice after OP treatment (Fig. 5L, M). The levels of pyroptotic markers (NLRP3, Caspase-1 and IL-18) and inflammation marker (ICAM-1) in aortic roots also displayed remarkable decrease after OP treatment (Fig. S9A–H). Collectively, these results suggested that administration of OP was a promising auxiliary medication for ASCVD treatment.

## Discussion

**Fig. 8:**
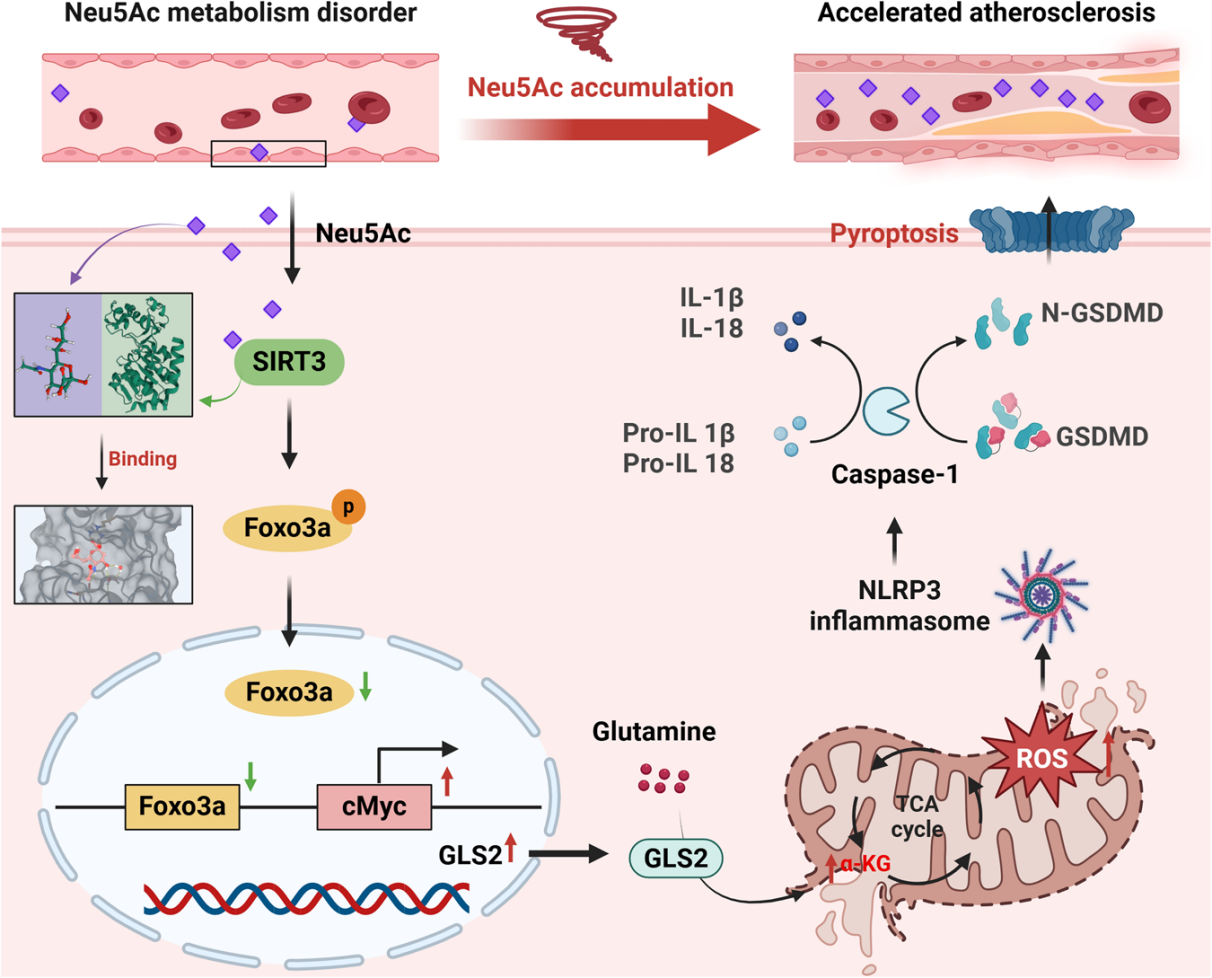
GLS2-mediated glutaminolysis accelerated AS process by encouraging endothelial mitochondrial dysfunction and pyroptosis via SIRT3/FOXO3a axis. Excess endogenous and exogenous Neu5Ac promoted the expression of transcriptional factor c-Myc via SIRT3/FOXO3a signaling pathway, contributing to increased transcription of GLS2 and accelerated glutaminolysis. With increased α-KG production and decreased GSH, Neu5Ac ultimately leads to mitochondrial damage, mitoROS production and pyroptosis in ECs and exacerbates atherosclerosis.

Despite the successful achievement in reducing the prevalence of ASCVD, the morbidity and mortality rates still remain high. Therefore, the identification of targets for halting the progression of AS represents a major challenge. In the present study, we highlighted the role of circulating metabolite, Neu5Ac, in pro-atherosclerosis process and updated its potential mechanism in promoting AS development. We confirmed that the elevated Neu5Ac was highly associated with AS progression as we reported before [14, 15]. Furthermore, we found that Neu5Ac induced mitochondrial dysfunction through NLRP3-associated pyroptosis, and in turns promoted ECs damage and AS progression. Mechanistically, Neu5Ac activated GLS2 via SIRT3/FOXO3a/c-Myc pathway, in turn lead to elevated α-KG and ROS as well as decreased GSH. These changes further contributed pyroptosis activation in vivo and in vitro. Inhibition of GLS2 or Neu5Ac accumulation should be a promising strategy for AS treatment (Fig. 8).

Neu5Ac is typically found in the terminal monosaccharides of glycans on cell surface. As acknowledged, Neu5Ac could be metabolized rapidly in body [46, 47]. And the elevated Neu5Ac in circulation was reported to be highly linked to various diseases including cancers and diabetes et al. [12, 47, 48]. Our previous work has administered Neu5Ac (60 mg/kg/d) to mice and further found that Neu5Ac acted as a novel inducer for AS progression through inducing endothelial dysfunction [14, 15]. However, the delivery methods and concentration we used for Neu5Ac differ with some studies. Yida et al. fed rat with free Neu5Ac (400 mg/kg/d) by gavage to study its effect on hypercoagulation pathway [49]. Yida et al. intraperitoneally injected rat with Neu5Ac twice daily (20 mg/kg twice a day) and Suzzi et al. subcutaneously administered to mice in a dose of 500 mg/kg twice a day [50]. Taken together, these studies further illustrated that the concentrations of supplementing exogeneous Neu5Ac were diverse. In our previous study, we noticed that the concentration detected in plasma may not represent the concentration which actually works [14, 15, 51]. However, Neu5Ac concentration in mice plasma showed a proportional increasing trend compared with the levels in patient plasma. Therefore, we used exaggerated concentrations of Neu5Ac, the similar experimental strategy with previous study, to better understand its role on endothelial dysfunction [7]. The results extend the understanding of Neu5Ac-associated endothelial dysfunction, and demonstrated the potential role of decreasing Neu5Ac for preventing AS progression. NEUs are partly responsible for cleaving terminal sialic acid residues from glycoproteins and oligosaccharides, the inhibitors of NEUs seems to present as potential target for AS treatment [52]. Here, we also confirmed that Oseltamivir, a widely used antiviral drug, effectively inhibits mammalian NEU enzymes and inhibited AS development, which was consistent with the previous study [7].

Endothelial dysfunction is the initial factor for early plaque formation [14, 15]. Recent studies have shown that pyroptosis is strongly involved in the initiation, the progression and complications of AS [53]. Oxidative stress, as we known, plays an important role in pyroptosis activation and endothelial damage induction [54]. Previous reports have identified that the NLRP3-associated pyroptosis pathway was activated by ROS, and ROS scavenger (NAC) could prevent endothelial cell pyroptosis [18]. In our current study, excessive ROS induced by Neu5Ac exposure also served as a priming stimulus to enhance the expression of NLRP3 inflammasome, ultimately promoting pyroptosis. Constient with the previous study [18], we also observed that NAC could protect ECs pyroptosis against ROS production, which further indicated the importance of mitochondrial homeostasis in regulating pyroptosis activation responsible for Neu5Ac stimulation. Furthermore, SIRT3/FOXO3a axis was reported to regulate mitochondrial homeostasis as well as ROS production [55]. FOXO3a can specifically suppress the expression of nuclear-encoded mitochondrial genes by antagonizing c-Myc, a known promoter of mitochondrial gene transcription [56]. And SIRT3 appears to have beneficial effects on activation of FOXO3a through deacetylation [57, 58]. Here, we further confirmed that Neu5Ac could bind to SIRT3 stably and induced phosphorylate its target, FOXO3a, for further nuclear translocation and functional antagonism to c-Myc. This cascade thereby heightened mtROS generation, and induced ECs dysfunction. Then, our results released signals that targeting SIRT3/FOXO3a/c-Myc pathway might be active for inhibiting pyroptosis induced by Neu5Ac, which still needs further investigation.

In addition, glutaminolysis was considered as a crucial factor for mitochondria homeostasis, and GLS2 was found to be strongly involved in regulating the conversion of glutamine to glutamate in mitochondria [27]. As acknowledged, both glutamine and glutamate were essential for GSH synthesis, and α-KG production can further induce pyroptosis by elevating ROS levels [59]. Previous study has found that elevated GLS2 could increase ROS production by facilitating the conversion of glutamate to α-KG, thereby promoting ferroptosis in hepatocellular carcinomas [47]. In our previous study, we also found that Neu5Ac triggered ferroptosis pathway and induced ROS production during AS progression [14], suggesting that GLS2 pathway might involve in the mitochondrial dysfunction induced by Neu5Ac. Here, we further confirmed that Neu5Ac increased GLS2 expression and α-KG levels, accompanied with a substantial accumulation of mtROS. Genetic deletion or pharmacological inhibition of GLS2 could markedly improve mitochondrial function, temper pyroptosis and attenuate atherosclerotic changes. These findings together highlighted the essential role of ROS in GLS2-associated mitochondrial dysfunction, also indicated the multiple roles of Neu5Ac in inducing endothelial injury. It is worth noting that, contrary to previous reports suggesting an antioxidant role for GLS2 via GSH [60], we found Neu5Ac decreased GSH levels in endothelial cells. This discrepancy may be partly attributed to ferroptosis pathway as we detected before [14], and also indicated that GLS2 might be a considerable target for regulating both ferroptosis and pyroptosis pathway responsible for Neu5Ac accumulation.

Nevertheless, there are several limitations in our present work. First, the present study mainly focus on the ECs and two kinds of endothelial cell lines including HUVEC and EA.hy926 were employed to observe the mechanism after Neu5Ac stimulation. We may further investigate the possible effect of Neu5Ac on macrophage and smooth muscle cells in our future work. Second, we observed an increase in GLS2 expression following Neu5Ac treatment, together with increased α-KG levels and decreased GSH levels. However, the effect on glutamine metabolism following Neu5Ac treatment requires further investigation. Third, our results showed that Neu5Ac bind to SIRT3 and then activate the FOXO3a/c-Myc/GLS2 signaling pathway. It’s plausible that other signaling pathways or transcription factors may also be involved in the regulation of GLS2, which requires further investigation. Finally, our present study demonstrated the potential anti-atherosclerotic effects of oseltamivir in animal models, clinical trials are needed to confirm its efficacy for AS treatment in future.

## Conclusions

This work identifies Neu5Ac as a crucial predisposing factor for atherosclerosis progression. Neu5Ac could promote GLS2-mediated glutaminolysis, and increase mitochondrial damage and mtROS production, thereby led to pyroptosis in ECs. Inhibiting Neu5Ac by targeting NEUs or GLS2 inhibition opens new fields for AS treatment, and also provide new insights into developing multipronged pharmacological interventions for ASCVD.

## Methods

### Mice

All animal experiments were followed the guidelines of the Institutional Animal Care and Use Committee of Chongqing Medical University (Approval Numbers: IACUC-CQMU-2022-0003). Totally, 52 ApoE^−/−^ mice (male, 8 weeks old) were generated on the C57BL/6j background, and they were purchased from Beijing Vital River Laboratory Animal Technologies Co., Ltd. Then, the mice were housed in a sterile environment under a 12 h/12 h light/dark cycle with suitable temperature (25 ± 2 °C) and humidity (50–60%). All mice had ad libitum access to a standard chow diet (Research Diets, D10001, medicience, China) or western diet (Research Diets, MD12015, medicience, China). In our study, 20 ApoE^−/−^ mice were fed on a normal chow diet and randomized into two groups which injected with normal saline or Neu5Ac (60 mg/kg, Aladdin Biochemical Technology Co., Ltd., Shanghai, China) twice a week for 8 weeks first and followed once a day for 4 weeks. After 12 weeks, all mice were sacrificed, aorta was extracted to detect serum lipid levels, atherosclerotic lesions and so on. The remaining ApoE^−/−^ mice were randomized into four groups (*n* = 8/group): control group, western diet group, lentiviral vector expressing GLS2 shRNA (HBLV-GLS2 shRNA, Hanbio Biotechnology Co. Ltd., Shanghai, China) group and oseltamivir phosphate group. The remaining group was fed on western diet, while the control group was fed on normal chow diet. Mice were injected with HBLV-NC or HBLV-GLS2 shRNA respectively (2 × 10^9^ TU/mL, interference sequence: GAACCTGCTATTTGCTGCATA) via tail vein twice after western diet started. Mice administered OP (10 mg/kg/day) effectively blocked mammalian NEU activity in vivo [61]. In our present study, oseltamivir phosphate (Rhawn Biochemical Technology Co., Ltd., Shanghai, China) was prepared in sterile water and administered at 10 mg/kg/day by oral gavage for 14 days before execution. Mice received sterile water were used as control. Twelve weeks later, 4% chloral hydrate and 3% sodium pentobarbital anesthesia were administered, and the mice were sacrificed to collect the heart, aorta and blood for further experiments. Specially, the further staining analysis was blinding.

### Materials and plasmids

Neu5Ac, Z-VAD-FMK (Caspase-1 inhibitor), necrostatin-1 (Nec-1), MCC950 (NLRP3 inhibitor), Compound 968 (GLS2 inhibitor), 10058-F4 (c-Myc inhibitor) and 3-TYP (SIRT3 inhibitor) were obtained from MedChemExpress (MCE); NAC (ROS scavenger) was obtained from Beyotime Biotechnology; oseltamivir phosphate was obtained from Rhawn Biochemical Technology Co., Ltd., Shanghai, China; DM-KG (α-KG analog) was from Aladdin Biochemical Technology; and LC/MS grade *α*-ketoglutarate and glutamine were from Zhongke Quality Inspection Biotechnology. Fetal bovine serum (FBS) was purchased from Biological Industries (Israel).

The plasmids utilized in the experiment were constructed by standard molecular cloning techniques. The lentiviral vectors carrying a shRNA for GLS2 (shGLS2) and a negative control shRNA (shControl) were designed and chemically synthesized by Hanheng Biotechnology Limited Company (Shanghai, China). The GLS2 target sequence is GAACCTGCTATTTGCTGCATA.

### Cell culture and transfection

EA. hy926 cells, HUVECs and THP-1 cells were grown in monolayer at 37 °C in 5% CO_2_. EA.hy926 cells were obtained from BeNa Culture Collection (China), and HUVECs were purchased from ScienCell Research Laboratories, Inc. (USA). EA.hy926 cells and HUVECs were maintained in DMEM supplemented with 10% FBS and 1% penicillin/streptomycin. Human acute monocytic leukemia cells (THP-1 cells) were purchased from BeNa Culture Collection (China) and cultured in RPMI 1640 medium supplemented with 10% fetal calf serum and 1% penicillin/streptomycin. Glutamine depletion medium was purchased from Procell Life Science & Technology and supplemented with 10% FBS and 1% penicillin/streptomycin. GLS2 and SIRT3 siRNA transfection was performed according to the manufacturer’s instructions. Cells were seeded on 12-well culture plates and transfected with the siRNA duplexes at ~70–80% confluence with Lipofectamine 2000 transfection reagent (Invitrogen, Carlsbad, CA, USA) according to the manufacturer’s instructions. Cells transfected with the control siRNA were treated in parallel. After 6 h of transfection, the medium was replaced with fresh medium for 36 h and then treated with or without Neu5Ac (20 mM) for 12 h. The cells were collected for protein/RNA extraction after drug treatment.

### Cytotoxicity assay

For cytotoxicity assays, HUVECs and EA.hy926 cells were seeded in 96-well plates, followed by drug treatment. CCK-8 solution was added to each well. After incubation for 3 h, absorbance was measured at 450 nm on a microplate reader.

### Propidium iodide (PI) staining

Cells were seeded in 24-well plates and treated as indicated. Cell culture supernatants were collected, and the cells were washed twice with PBS. Then, the cells were harvested by trypsin enzymatic digestion and mixed with the supernatants collected before. After centrifugation, Hoechst staining (5 μg/mL) and PI (5 ng/mL) solution were added to the cells for 30 min at 37 °C in a dark environment. Then, the cells were washed twice again with PBS, plated in 24-well plates and maintained at 37 °C with 5% carbon dioxide (CO_2_) for 20 min. The dead cells were observed under a fluorescence microscope at room temperature.

### Immunoblotting

Cells or tissues were collected and homogenized in RIPA buffer supplemented with 1% phenylmethanesulfonyl fluoride. The protein concentrations of the lysates were measured with a BCA assay kit (Beyotime, China). Equal amounts of total proteins were separated by SDS–PAGE and transferred to PVDF membranes. Immunoreactivity was detected using a Pierce ECL kit (Biosharp, China). The primary antibodies used for immunoblots were as follows: Rabbit Polyclonal Antibody against IL-1β (Beyotime, AF7209, diluted 1:1000), Rabbit Monoclonal Antibody against NLRP3 (Beyotime, AF2155, diluted 1:1000), Rabbit Monoclonal Antibody against Caspase-1 (Beyotime, AF1681, diluted 1:1000), Rabbit Monoclonal Antibody against VCAM-1 (Beyotime, AF1021, diluted 1:1000), Rabbit Polyclonal Antibody against ICAM-1 (Beyotime, AF0195, diluted 1:1000), Rabbit Polyclonal Antibody against GLS2 (ABclonal, A16029, diluted 1:1000), Rabbit Polyclonal Antibody against GSDMD (full length + N-terminal) (ABclonal, A20197, diluted 1:1000), Rabbit Polyclonal Antibody against GSDME (ABclonal, A7432, diluted 1:1000), Rabbit Polyclonal Antibody against IL-18 (ABclonal, A16737, diluted 1:1000), Rabbit Polyclonal Antibody against Phospho-FOXO3A-S253 (ABclonal, AP0684, diluted 1:1000), Rabbit Polyclonal Antibody against c-Myc (Proteintech, 10828-1-AP, diluted 1:1000), mouse Monoclonal Antibody against FOXO3a (Proteintech, 66428-1-Ig, diluted 1:1000), mouse Monoclonal Antibody against SIRT3 (Santa Cruz, sc-365175, diluted 1:1000). Horseradish peroxidase-conjugated goat anti-rabbit IgG (Beyotime, A0208, diluted 1:8000) and horseradish peroxidase-labeled goat anti-mouse IgG (Beyotime, A0216, diluted 1:8000) were used as the secondary antibodies.

### Immunofluorescence assay

EA.hy926 cells and HUVECs were plated on glass slides in 24-well plates and cultured overnight at 37 °C. After treatment, the cells were fixed with 4% paraformaldehyde for 15 min, and the cell membrane was permeabilized with 0.1% Triton X-100. Then, the cells were blocked with 5% bovine serum protein for 1 h at room temperature. After that, the cells were incubated with primary antibody at 4 °C overnight, followed by incubation with corresponding secondary antibodies for 1 h at room temperature. Ultimately, the cells were incubated with DAPI for 10 min and observed under a fluorescence microscope.

Fluorescence images of slices from aortic roots in mice were captured with a confocal laser scanning microscope after co-immunofluorescent staining. Z-stacks with three slices of 2 µm thickness were recorded. Single images from Z-stacks were combined into stacks and transformed into Z-projects with maximal intensities using LSM software [28].

### Intracellular ROS measurement

EA.hy926 cells were seeded in 6-well plates and treated as indicated. Cells were washed twice with PBS and harvested by trypsin enzymatic digestion. After centrifugation, the cells were resuspended in DCFH-DA solution (Beyotime, China, 10 μM) at 37 °C for 15 min in a dark environment. The intracellular ROS were measured by using flow cytometry.

### Mitochondrial ROS measurement

Briefly, cells were seeded on confocal dishes at ~70–80% confluence. After the indicated treatment, the cells were incubated with Mitosox (Beyotime, China, 5 μM) at 37 °C for 15 min in a dark environment, and the cells were carefully washed twice with cold PBS. Fluorescence images of mtROS were captured using a laser scanning confocal microscope at an excitation wavelength of 510 nm and emission wavelength of 580 nm.

### Mitochondrial mass measurement

Mitochondrial mass was determined by a MitoTracker Green probe (Beyotime, China). After treatment, the EA.hy926 cells and HUVECs were incubated with 80 nM MitoTracker for 30 min at 37 °C in a dark environment. Fluorescence images of the mitochondrial mass were captured using a laser scanning confocal microscope at an excitation wavelength of 490 nm and emission wavelength of 516 nm.

### Mitochondrial membrane potential measurement

Mitochondrial membrane potential was measured according to the manufacturer’s instructions. EA.hy926 cells and HUVECs were seeded on confocal dishes and concurrently treated with *N*,*N*,*N*′,*N*′-tetra-methylethylenediamine (TMRE) (Beyotime, China) for 1 h at 37 °C in a dark environment. Fluorescence images of the mitochondrial mass were captured using a laser scanning confocal microscope at an excitation wavelength of 550 nm and emission wavelength of 575 nm.

### Monocyte adhesion assay

EA.hy926 cells and HUVECs were seeded on 12-well plates. After treatments, THP-1 cells were labeled with Calcein AM (Beyotime, China) and incubated on top of a monolayer of HUVECs for 1 h. Cells were washed three times with PBS. Monocyte adhesion was observed using an Olympus inverted fluorescence microscope.

### RNA isolation and qRT-PCR

Total RNA from cells and tissues was extracted by TRIzol reagent. The purity and concentration of the extracted RNA were evaluated using a Nanodrop 3000 (ThermoFisher, Scotts Valley, CA, USA). An Eco M-MLV Mix Kit with gDNA Clean for qPCR (Accurate Biotechnol, China) was used to synthesize complementary DNA. qRT-PCR was performed using SYBR® Green Realtime Master qPCR Mix (TsingKe Biotech, China) on an ABI 7900HT Fast Real-Time PCR System (Applied Biosystems, Foster City, CA, USA). The levels of ACTIN or GAPDH mRNA were used as internal controls. The primers used are listed in Supplementary Table S1 and were synthesized by Tsingke Biotechnology Co., Ltd. (Beijing, China). The specificity of all PCR products was assessed by melting curve analysis. The relative mRNA expression values were calculated in accordance with the 2^−ΔΔCt^ method.

### Cell and mitochondrial morphology observation

After treatment, cells were fixed with phosphate-buffered glutaraldehyde. Cell morphology and mitochondrial morphology were then observed by transmission electron microscopy (Tecnai, G2 F30 S-TWIN, USA).

### Atherosclerosis plaque analysis

After euthanasia, the mice were perfused with saline through a left ventricle puncture. The heart and whole aorta were dissected from mice, and the adjoining tissues were removed. For assessment of the en face lesion area, the whole aorta was unfolded longitudinally and stained with Oil Red O. For analyses of atherosclerotic lesions in the aortic root, the upper portion of the heart and proximal aorta were separated carefully, and a series of 8 μm frozen sections were prepared and processed for Oil Red O, hematoxylin–eosin (HE), Masson staining, TUNEL staining and immunostaining of Caspase-1, NLRP3, IL-18, ICAM-1 and CD31 (endothelial cell marker) to detect cell death and activation of the aforementioned proteins in endothelial cells. Image-Pro Plus 7.0 software was used for quantitative analyses.

### Biochemical assays

Blood samples were collected from orbit and centrifuged at 7500 rpm for 15 min at 4 °C for various biochemical assays, such as aspartate transaminase, ALT, albumin, blood urea nitrogen and creatinine.

### α-KG measurement

Extracts were prepared from ~5 × 10^6^ cells with methanol solution according to the protocol described by Tetsushi Yamamoto [29]. The filtrate was centrifugally concentrated and dissolved in ammonium hydroxide immediately before LC/MS analysis.

### Determination of GSH, glutamate and glutamine concentrations

The concentrations of GSH, glutamate and glutamine in endothelial cells were determined by using a Glutathione Assay Kit (Beyotime, China), Glutamate Colorimetric Assay Kit (Elabscience, China) and Glutamine (Gln) Colorimetric Assay Kit (Elabscience, China), respectively, in accordance with the instructions.

### Study participants and measurement of plasma Neu5Ac in human

The human study was approved by the Ethics Committee of the University Town Hospital of Chongqing Medical University (Approval Numbers: NO.LL-202256). Enrollment included 17 patients with hyperlipemia and 17 matched normal individuals. None received lipid-lowering medications or were hospitalized. The information was collected during a routine examination, with written informed consent obtained from all participants in advance. Neu5Ac levels in plasma were quantified by a Sialic Acid (SA) Colorimetric Assay Kit (Elabscience Biotechnology Co., Ltd., China) according to the manufacturer’s instructions.

### Statistical analysis

Statistical comparisons between two independent groups were performed with Student’s *t-*test, while one-way ANOVA tests were used to evaluate significant differences between two conditions. GraphPad Prism 7 and SPSS 24.0 were used for data analysis. Data are presented as the average value ± SEM, and *p* values < 0.05 were considered statistically significant.

## Supplementary information

Supplemental figures and tables

Raw data

## Data Availability

Almost all data generated or analyzed during this study are included in this article and the Supplementary Information files, some unexposed data analyzed during the current study are available from the corresponding author on reasonable request.
