# Supplementary material for: N-Acetylneuraminic acid triggers endothelial pyroptosis and promotes atherosclerosis progression via GLS2-mediated glutaminolysis pathway
Source: Cell Death Discov. 2024 Nov 13;10:467. doi: 10.1038/s41420-024-02233-7 (PMC11561128; doi:10.1038/s41420-024-02233-7)
Supplement: Supplementary file 1 — Supplemental figures and tables [file 41420_2024_2233_MOESM1_ESM.pdf]

Supplemental Figures

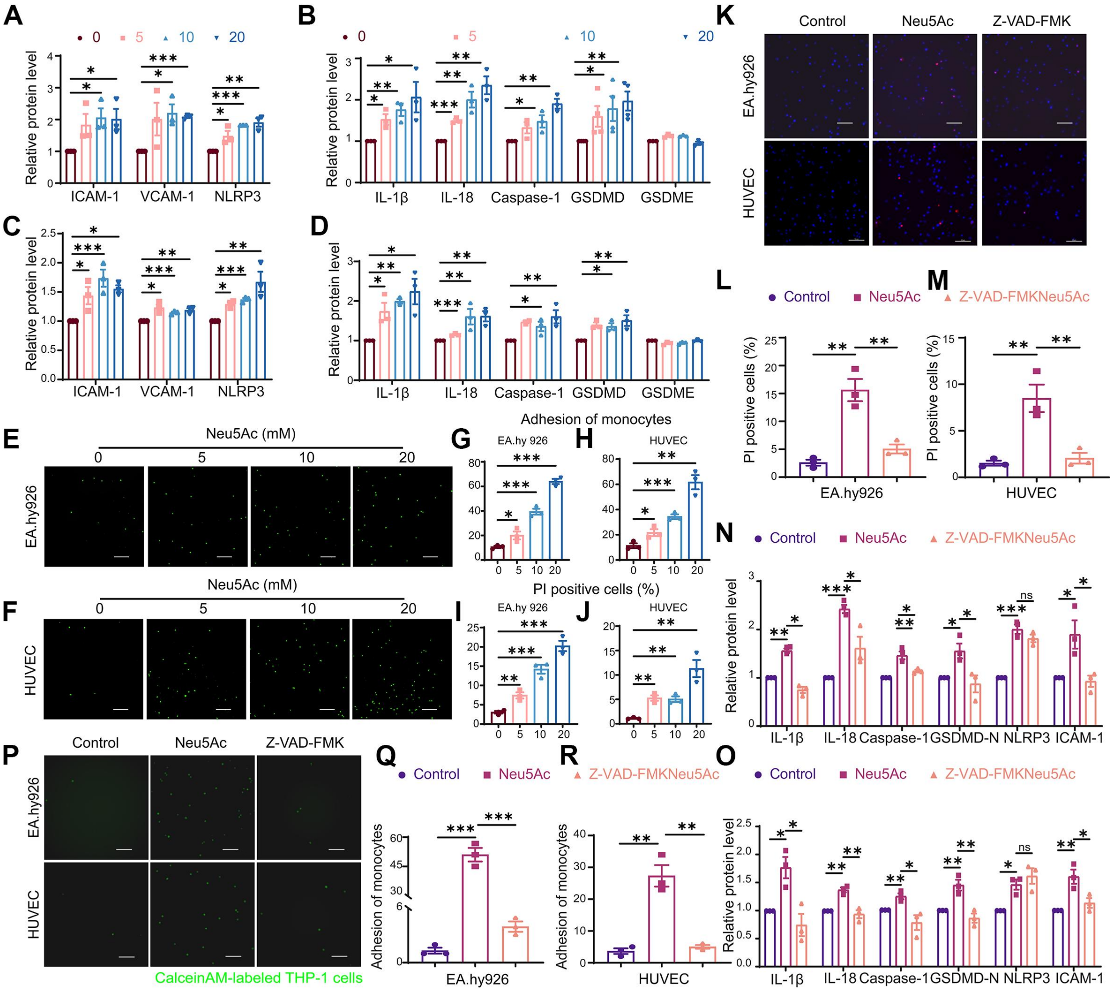

Figure S1. Neu5Ac induced endothelial inflammatory injury in pyroptosis dependent way in HUVECs and EA. Hy926 cells .

A-D, quantitative analysis of pyroptosis markers (GSDMD-N, GSDME, NLRP3 inflammasome, Caspase-1, IL-1β, and IL-18) and pro-inflammatory adhesion molecules (ICAM-1 and VCAM-1) protein expression in ECs treated with indicated concentrations of Neu5Ac for 12 h. **E-H**, Monocyte-endothelial adhesion analysis in ECs treated with indicated concentrations of Neu5Ac for 12 h, with quantitative data at below. Bar = 100 μm. **I and J**, quantitative analysis of PI-positive cells in ECs treated with indicated concentrations of Neu5Ac for 12 h. ECs were preincubated with or without Z-VAD-FMK (10 μM) for 1 h before Neu5Ac (20 mM) treatment for 12 h, Hoechst/PI staining and quantitative analysis of cell death were performed (**K - M**); quantitative analysis of GSDMD, NLRP3, Caspase-1, IL-1β, IL-18 and ICAM-1 protein expression (**N and O)**; Monocyte-endothelial adhesion analysis in ECs were performed and quantified. Bar = 100 μm (**P - R**). Data were analyzed using unpaired

two-tailed student t-tests or one-way ANOVA tests, and presented as the means ± SEM. * $p < 0.05$ was considered significant, ** $p < 0.01$, *** $p < 0.001$.

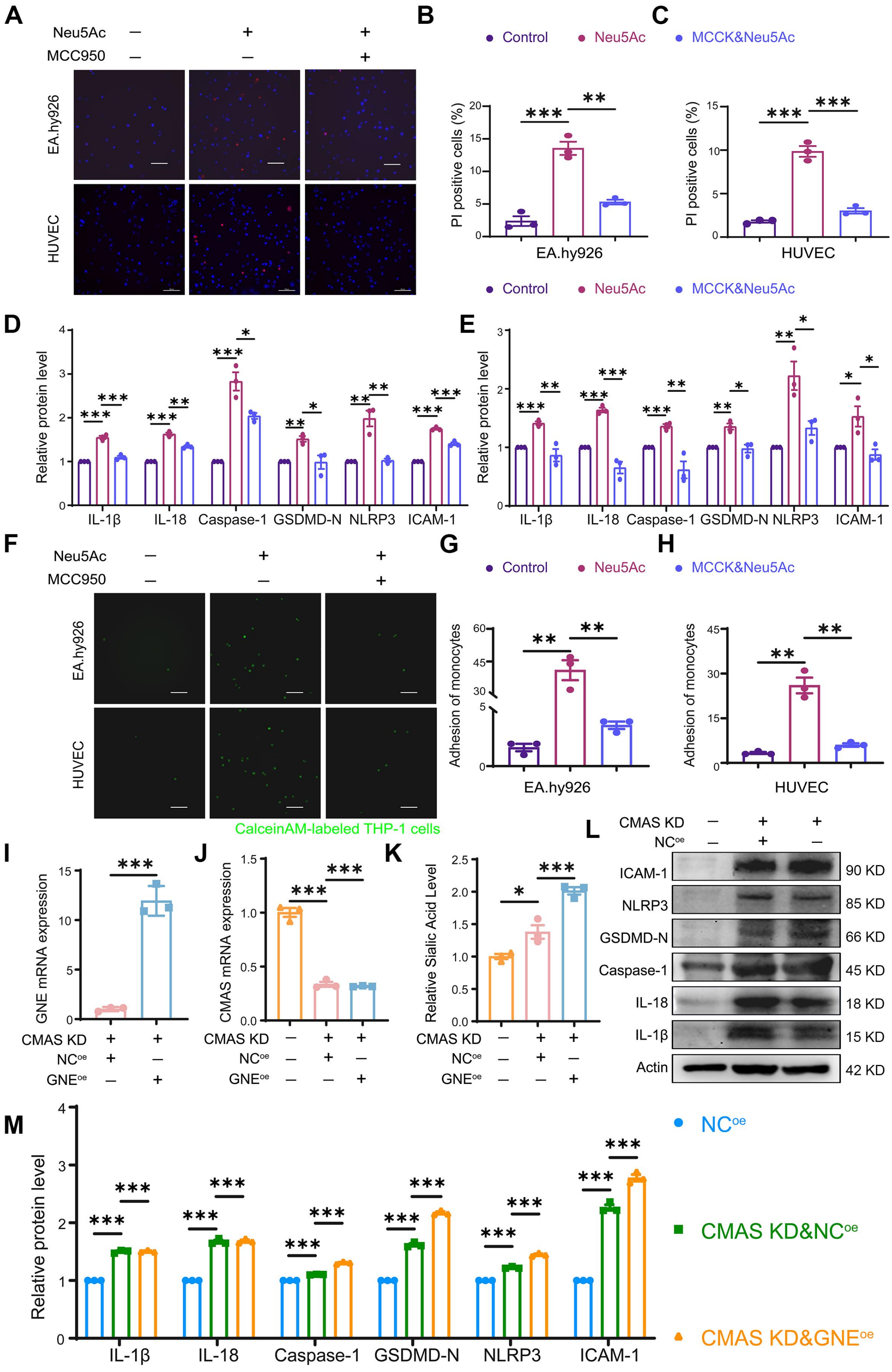

Figure S2. Endothelial pyroptosis injury was highly associated with elevated Neu5Ac levels *in vitro*.

A-H, ECs were preincubated with or without MCC950 (10 μM) for 1 h before Neu5Ac (20 mM) treatment for 12 h, Hoechst/PI staining and quantitative analysis of PI-positive cells in ECs (**A-C**); quantitative analysis of GSDMD-N, NLRP3, Caspase-1, IL-1β, IL-18 and ICAM-1 protein expression (**D and E**); Monocyte-endothelial adhesion analysis in ECswere performed and quantified. Bar = 100 μm (**F-H**). **I-K**, CMAS KD HUVECs were developed by lentivirus-mediated transfection of CMAS, GNE plasmid were then transfected in CMAS KD HUVECs, the efficiency of transfection were measured by qRT-PCR analysis (**I and J**); the sialic acid level in these transfected cells were measured by Biochemical Assay Kit (**K**); IL-1β, IL-18, Caspase-1, NLRP3, GSDMD-N and ICAM-1 protein expression were assessed by Western blot (**L**), with quantitative data at below (**M**). Data were analyzed using unpaired two-tailed student t-tests or one-way ANOVA tests, and presented as the means ± SEM. * $p < 0.05$ was considered significant, ** $p < 0.01$, *** $p < 0.001$.

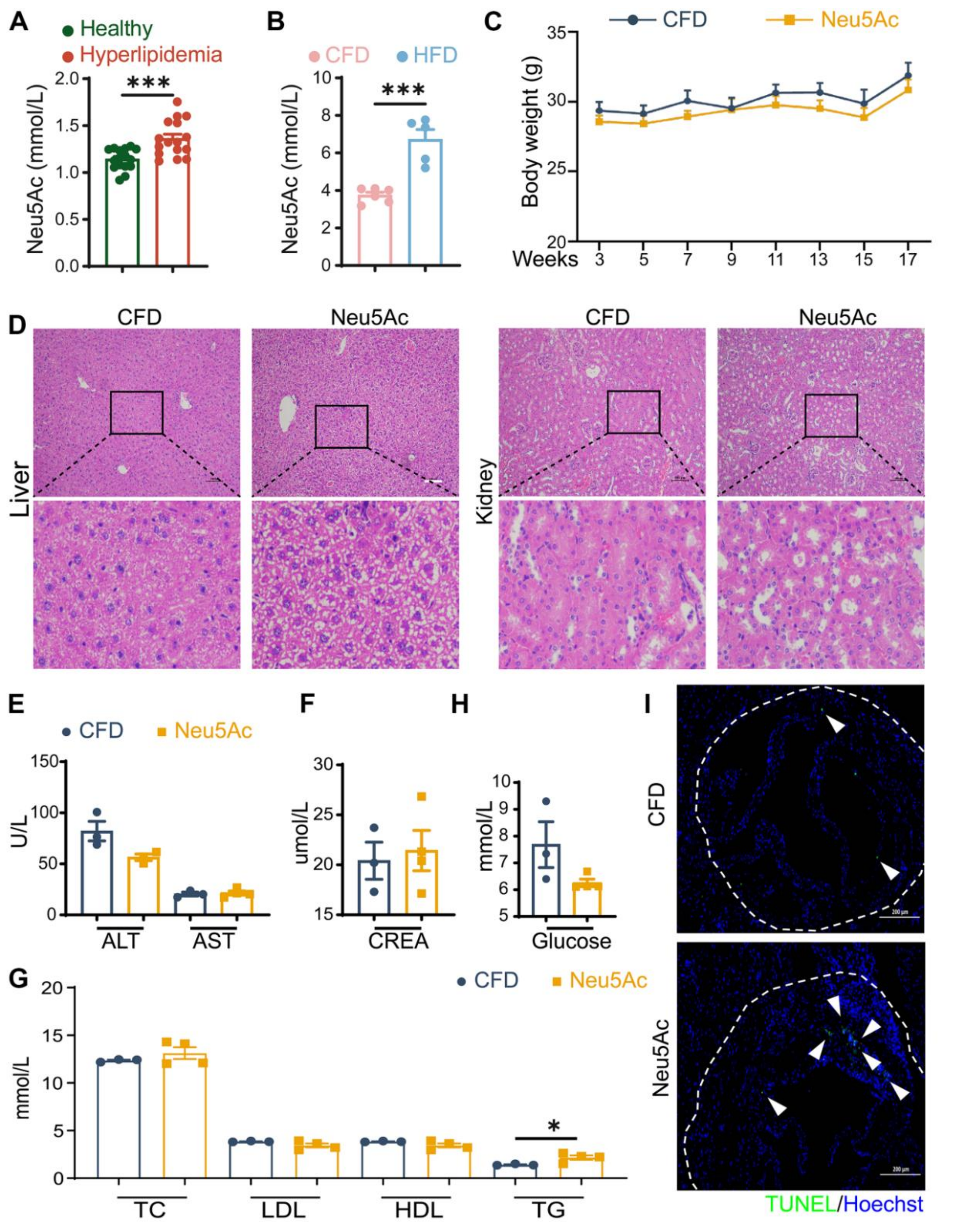

Figure S3. AS progression was highly associated with Neu5Ac levels *in vivo*.

A, Comparison of Neu5Ac level in blood serum between hyperlipidemia patients (n=17) and healthy donors (n=17). **B**, Comparison of Neu5Ac level in blood serum between HFD-fed atherosclerotic ApoE$^{-/-}$ mice (n=5) and CFD-fed ApoE$^{-/-}$ mice (n=5). **C**, The body weight of ApoE$^{-/-}$ mice injected with 0.9% N.S. or Neu5Ac. **D**, Hematoxylin and eosin (HE) staining of Liver and Kidney from ApoE$^{-/-}$ mice injected with 0.9% N.S. (n=3) or Neu5Ac (n=4). Bar = 100 μm. **E-H**, biochemical analysis

showing the level of ALT, AST, CREA, Glucose, total cholesterol, LDL cholesterol, HDL cholesterol and triglyceride in the serum of $ApoE^{-/-}$ mice injected with 0.9% N.S. or Neu5Ac. **I,** TUNNEL staining of atherosclerotic lesions of aortic roots in $ApoE^{-/-}$ mice injected with 0.9% N.S. (n=3) or Neu5Ac (n=3). Data were analyzed using unpaired two-tailed student t-tests or one-way ANOVA tests, and presented as the means ± SEM. * $p < 0.05$ was considered significant, ** $p < 0.01$, *** $p < 0.001$.

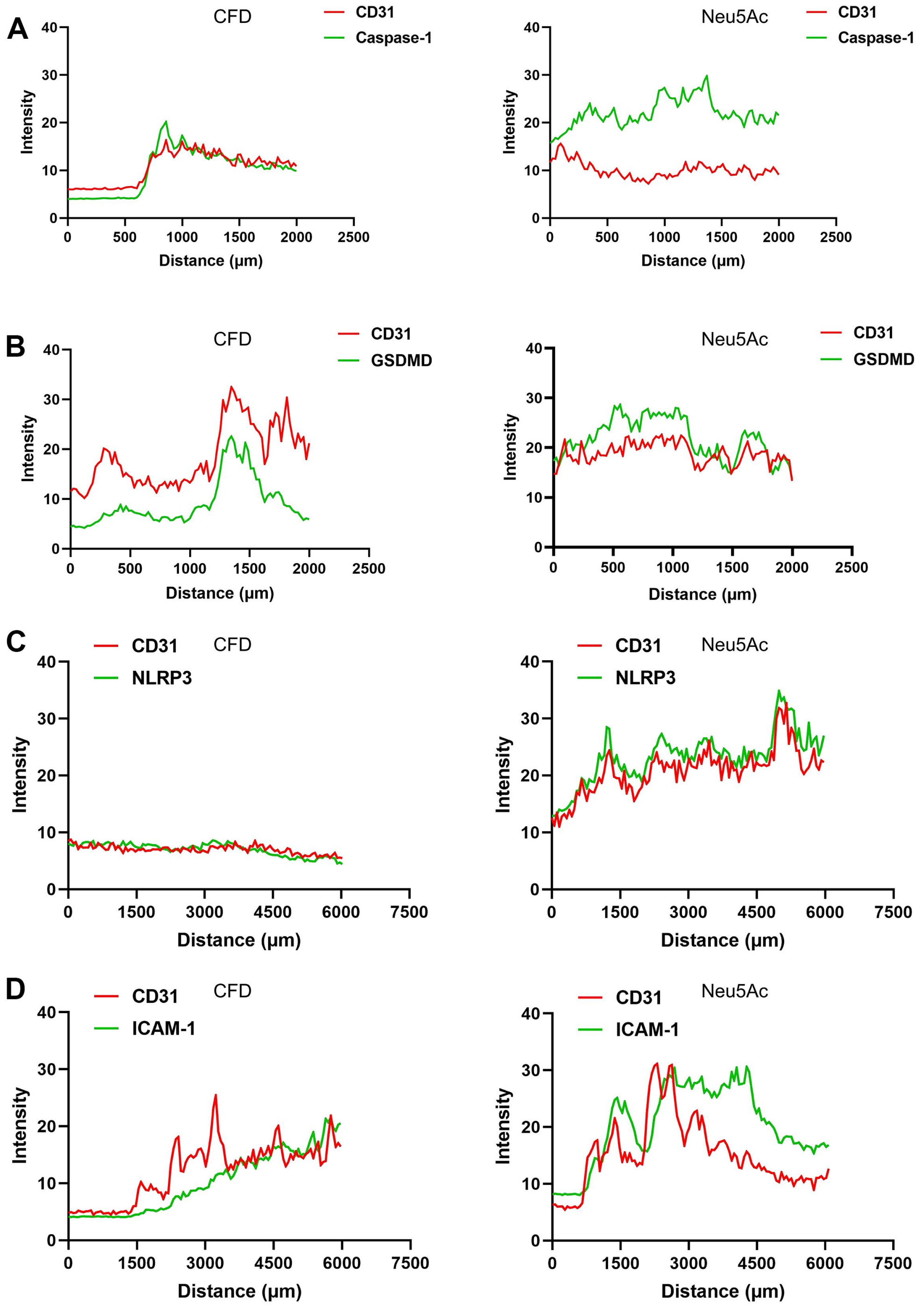

Figure S4. Analysis of pyroptosis as well as inflammatory makers in mice after Neu5Ac treatment.

A-D, quantitative analysis of the images from Caspase-1, GSDMD, NLRP3 and ICAM-1 co-immunoflurorescence staining with CD31 of aortic root from ApoE$^{-/-}$ mice injection with 0.9% N.S. (n=3) or Neu5Ac (n=3). Bar = 100 μm.

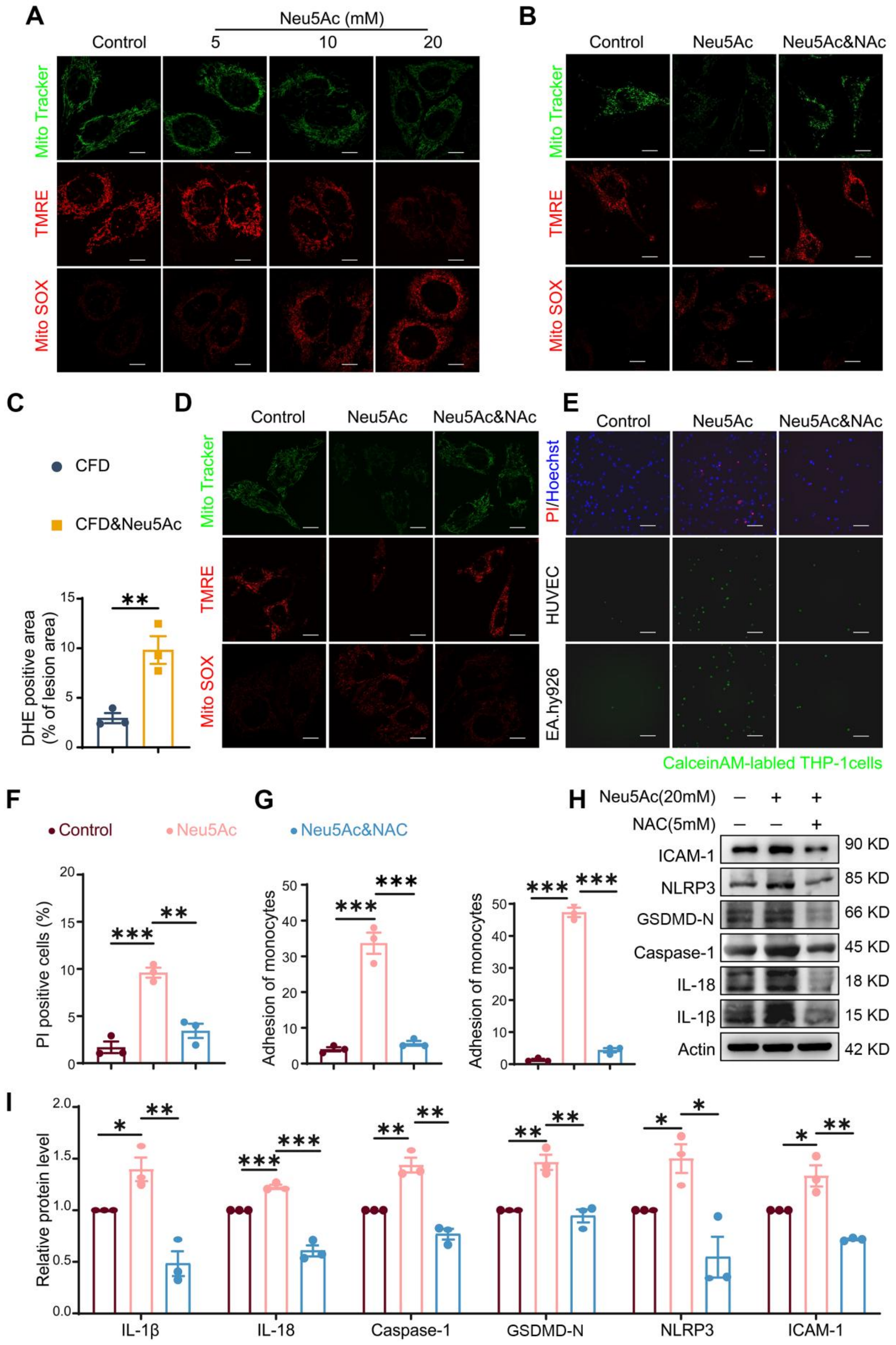

Figure S5. Neu5Ac increased mitochondrial ROS and activated pyroptosis pathway

A, HUVEC were treated with indicated concentrations of Neu5Ac for 12 h, mitochondrial mass was labeled by MitoTracker Green; the mitochondrial membrane potential was assayed by TMRE; The mitochondrial ROS level were detected by mitoSOX. Bar = 31.75 µm. **B**, quantitative analysis of the images of DHE staining in aortic root from 0.9% N.S. (n=3) or Neu5Ac-injected $ApoE^{-/-}$ mice (n=3). **C and D**, EA.hy926 cells and HUVEC were preincubated with or without NAC (5 mM) for 1 h before Neu5Ac (20 mM) treatment for 12 h, mitochondrial mass was labeled by MitoTracker Green; the mitochondrial membrane potential was assayed by TMRE; The mitochondrial ROS level were detected by mitoSOX. Bar = 31.75 µm. **E-I**, ECs were preincubated with or without NAC (5 mM) for 1 h before Neu5Ac (20 mM) treatment for 12 h, cell death in HUVEC were detected by PI staining and quantified (**E and F**); Monocyte-endothelial adhesion analysis in ECs was performed and quantified (**E - G**). Bar = 100 µm; IL-1β, IL-18, Caspase-1, NLRP3, GSDMD-N and ICAM-1 protein expression in HUVEC were assessed by western blot, with quantitative data at right (**H and I**). Data were analyzed using unpaired two-tailed student t-tests or one-way ANOVA tests, and presented as the means ± SEM. * $p < 0.05$ was considered significant, ** $p < 0.01$, *** $p < 0.001$.

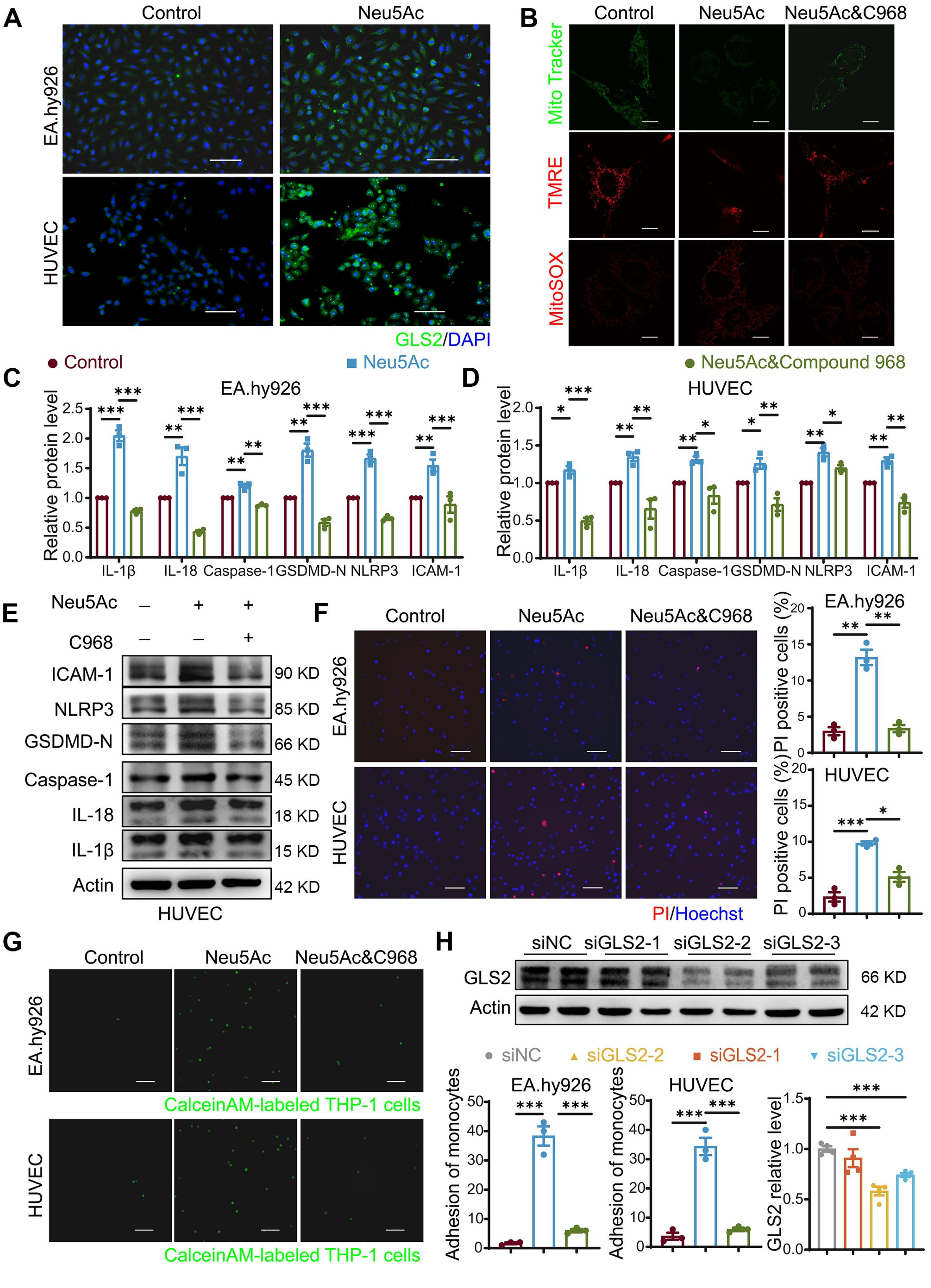

Figure S6. GLS2-mediated glutaminolysis facilitated pyroptosis in ECs.

A, GLS2 and DAPI immunostaining in ECs treated with or without Neu5Ac (20 mM). Bar = 100 μm. **B and C**, EA.hy926 cells were preincubated with or without Compound 968 (10 μM) for 1 h before Neu5Ac (20 mM) treatment for another 12 h,

mitochondrial mass, membrane potential and mitochondrial ROS production in were labeled. Bar = 31.75 μm (**B**); quantitative analysis of IL-1β, IL-18, Caspase-1, NLRP3, GSDMD-N and ICAM-1 protein expression (**C**); **D and E**, HUVEC were preincubated with or without Compound 968 (10 μM) for 1 h before Neu5Ac (20 mM) treatment for another 12 h, IL-1β, IL-18, Caspase 1, NLRP3, GSDMD-N and ICAM-1 protein expression were analyzed by WB assay and quantified. **F and G**, ECs were preincubated with or without Compound 968 (10 μM) for 1 h before Neu5Ac (20 mM) treatment for another 12 h, cell death were labeled by Hoecchst/PI staining and quantified (**F**); Monocyte-endothelial adhesion analysis were performed and quantified (**G**); Bar = 100 μm. **H**, EA.hy926 cells were tranfected with siGLS2-1, siGLS2-2 and siGLS2-3, GLS2 protein expression were analyzed by western blot, with quantitative data at below. Data were analyzed using unpaired two-tailed student t-tests or one-way ANOVA tests, and presented as the means ± SEM. * $p < 0.05$ was considered significant, ** $p < 0.01$, *** $p < 0.001$.

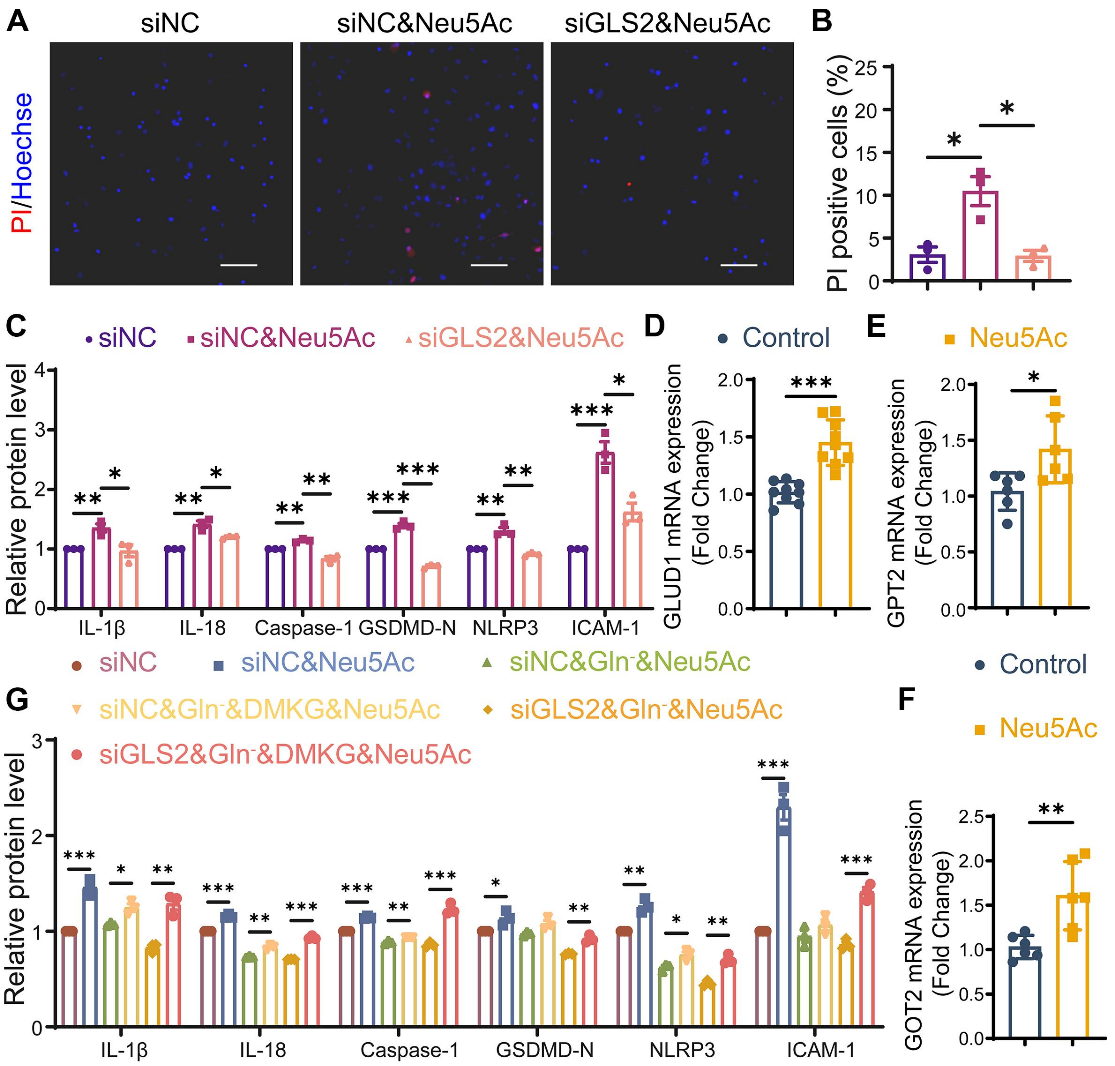

Figure S7. siGLS2 in ECs inhibited pyroptosis associated inflammatory injury.

A-**C**, EA.hy926 cells were tranfected with GLS2 siRNA before Neu5Ac (20 mM) treatment for 12 h, cell death were labeled by Hoecchst/PI staining and quantified (**A and B**); quantitative analysis of IL-1β, IL-18, Caspase-1, NLRP3, GSDMD-N and ICAM-1 protein expression (**C**); **D-F**, qRT-PCR analysis of GLUD1, GPT2 and GOT2 in Neu5Ac-treated EA.hy926 cells; **G**, quantitative analysis of IL-1β, IL-18, Caspase-1, NLRP3, GSDMD-N and ICAM-1 protein expression of EA.hy926 cells deprived of Gln (-Gln) or transfected with GLS2 siRNA followed by Neu5Ac (20 mM) treatment for 12h in the presence or absence of 4 mM DMKG for 3 h. Data were analyzed using unpaired two-tailed student t-tests or one-way ANOVA tests, and presented as the means ± SEM. * $p < 0.05$ was considered significant, ** $p < 0.01$, *** $p < 0.001$.

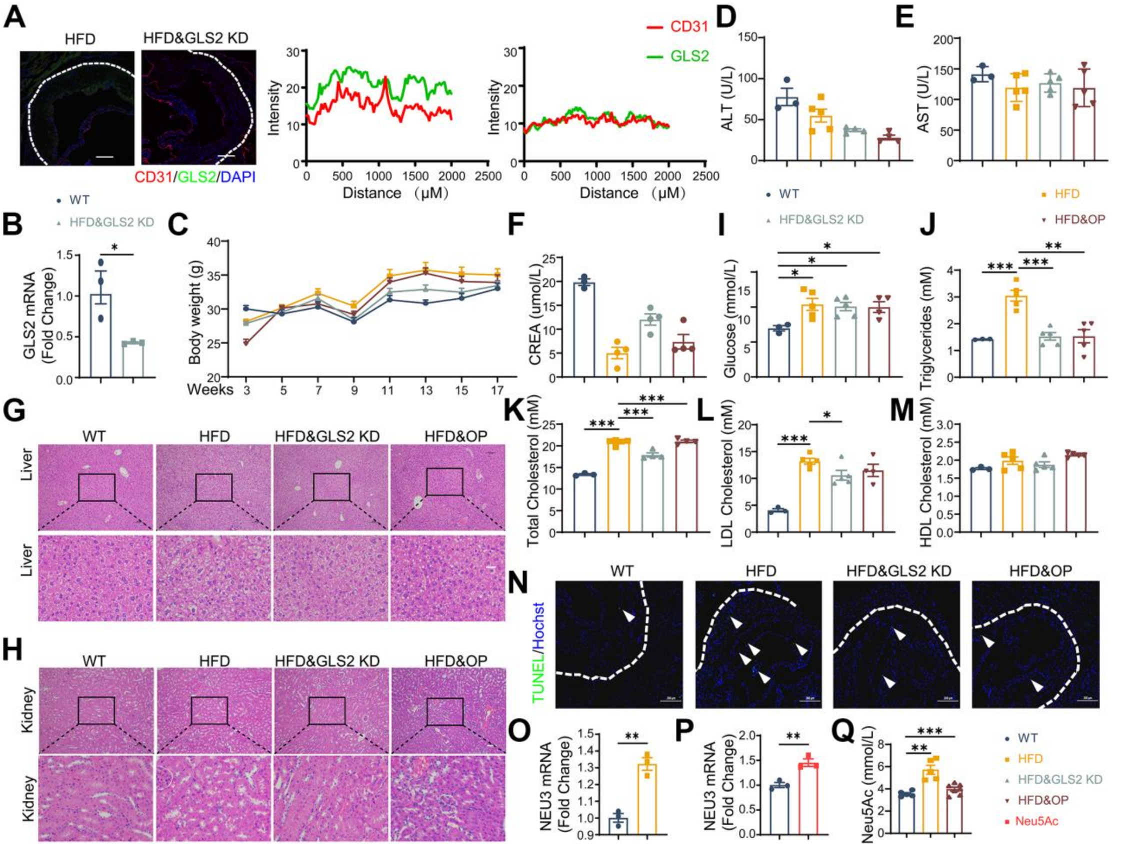

Figure S8. GLS2 knockdown in ApoE$^{-/-}$ mice mitigated atherosclerotic changes.

A, CD31 and GLS2 immunostaining of aortic root from HFD and GLS2 KD ApoE$^{-/-}$ mice, with quantitative data at right. Bar = 100 μm. **B**, qRT-PCR analysis of GLS2 expression in aortic artery from HBLV-shControl or HBLV-shGLS2 injected ApoE$^{-/-}$ mice. **C**, The body weight of WT, HFD, GLS2 KD and OP-administrated ApoE$^{-/-}$ mice. **D-F**, biochemical analysis showing the level of AST, ALT and CREA in the serum of Control, HFD, HBLV-shGLS2 and OP-administrated ApoE$^{-/-}$ mice. **G-H**, HE staining of Kidney and Liver from WT, HFD, GLS2 KD and OP-administrated ApoE$^{-/-}$ mice. Bar = 100 μm. **I-K**, biochemical analysis showing the level of triglyceride, total cholesterol, LDL cholesterol, HDL cholesterol and glucose in the serum of WT, HFD, GLS2 KD and OP-administrated ApoE$^{-/-}$ mice. **L**, TUNEL staining showed cell death in aortic root from WT, HFD, GLS2 KD and OP-administrated ApoE$^{-/-}$ mice. Bar = 100 μm. **M**, qRT-PCR analysis of NEU3 in aortic artery of CFD and HFD fed ApoE$^{-/-}$ mice. **N**, qRT-PCR analysis of NEU3 in aortic artery of 0.9% N.S. or Neu5Ac-injected ApoE$^{-/-}$ mice. **O**, comparison of Neu5Ac level in blood serum in CFD, HFD and OP-administrated ApoE$^{-/-}$ mice. Data were analyzed using unpaired

two-tailed student t-tests or one-way ANOVA tests, and presented as the means ± SEM. * $p < 0.05$ was considered significant, ** $p < 0.01$, *** $p < 0.001$.

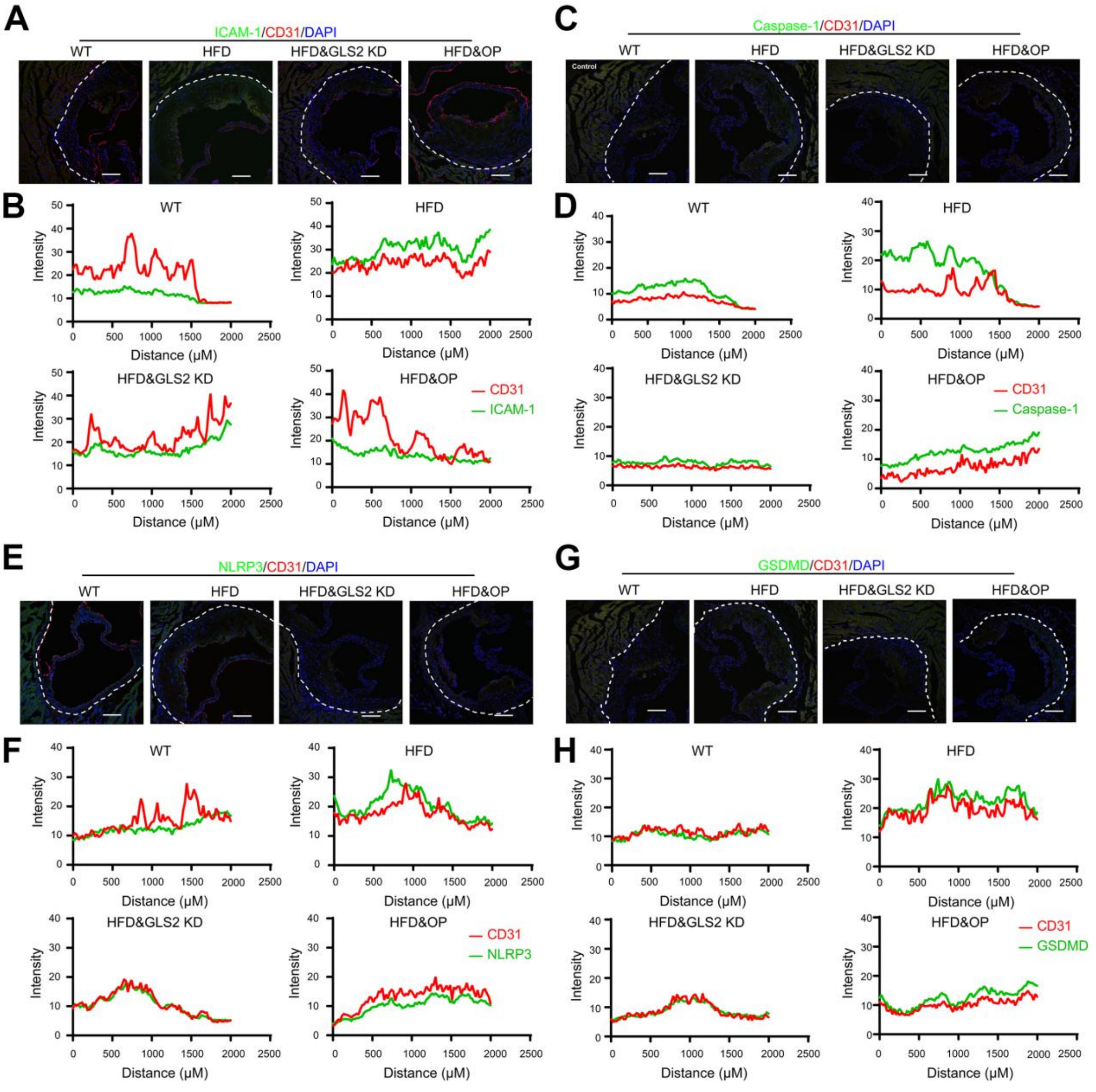

Figure S9. Pyroptosis as well as inflammatory makers in aortic root from GLS2 knockdown ApoE$^{-/-}$ mice.

A-H, Caspase-1, GSDMD, NLRP3 and ICAM-1 co-immunoflurorescence staining with CD31 of aortic root from ApoE$^{-/-}$ mice injection with 0.9% N.S. (n=3) or Neu5Ac (n=3), with quantitative data at below. Bar = 100 μm.

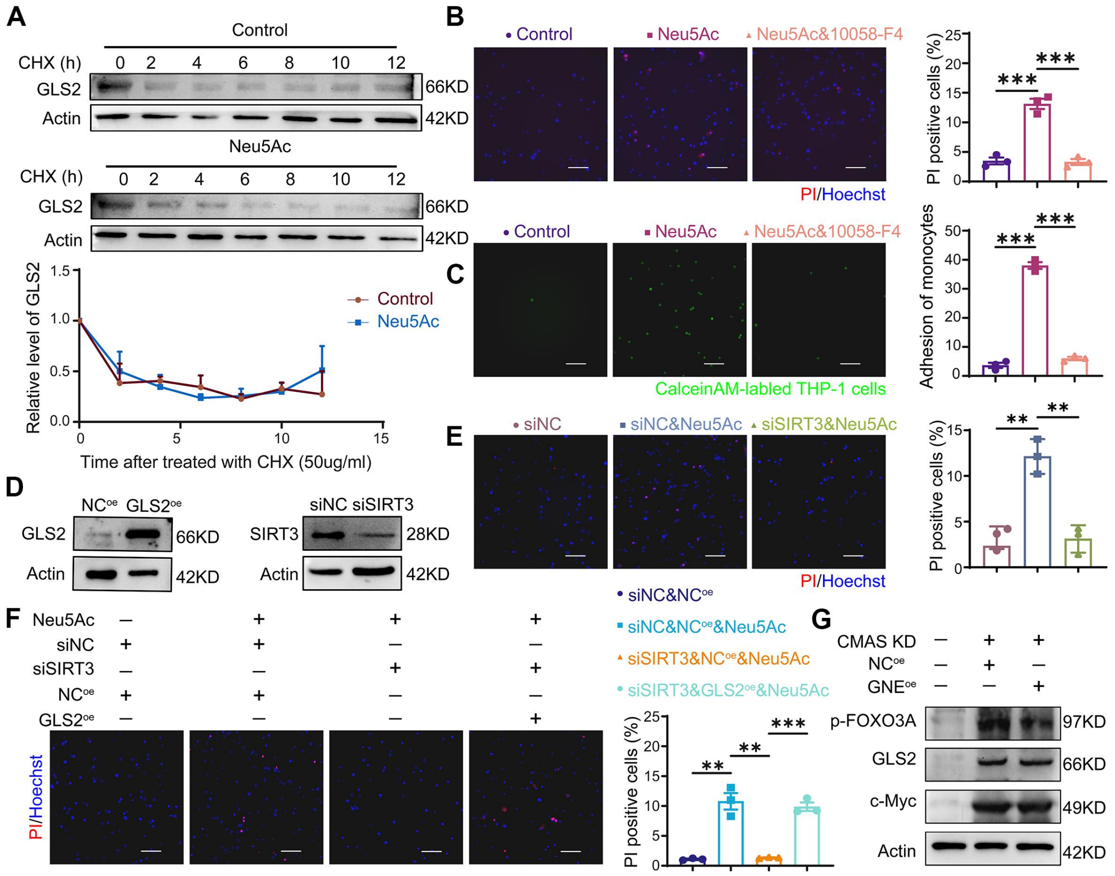

Figure S10. SIRT3/FOXO3a pathway participated in GLS2 activation induced by Neu5Ac. A, Protein stability of GLS2 was assessed by western blot analysis in Neu5Ac-treated EA.hy926 cells co-incubation with CHX (50 ug/ml) for indicated hours (2, 4, 6, 8, 10 and 12 h). **B-C**, EA.hy926 cells were pre-incubated with or without 10058-F4 (60 μM) for 1 h before Neu5Ac (20 mM) treatment for 12 h, cell death in EA.hy926 cells were labeled by Hoechst/PI staining and quantified (**B**); Monocyte-endothelial adhesion analysis were performed and quantified (**C**); Bar = 100 μm. **D**, EA.hy926 cells transfected with GLS2 plasmid and SIRT3 siRNA followed by Neu5Ac (20 mM) treatment for 12 h, the transfection efficiency were assessed by WB assay. **E**, EA.hy926 cells were transfected with SIRT3 siRNA or NC siRNA before Neu5Ac (20 mM) treatment for 12 h, cell death in EA.hy926 cells were labeled by Hoechst/PI staining and quantified . Bar = 100 μm. **F**, CMAS KD HUVECs were developed by lentivirus-mediated transfection of CMAS, GNE plasmid were then transfected in CMAS KD HUVECs, as the sialic acid level in these transfected cells were increased, the protein expression of p-FOXO3a, c-Myc and GLS2 were assessed by western blot. **G**, EA.hy926 cells transfected with GLS2

plasmid and SIRT3 siRNA followed by Neu5Ac (20 mM) treatment for 12 h, cell death in EA.hy926 cells were labeled by Hoechst/PI staining and quantified. Bar = 100 μm. Data were analyzed using unpaired two-tailed student t-tests or one-way ANOVA tests, and presented as the means ± SEM. * $p < 0.05$ was considered significant, ** $p < 0.01$, *** $p < 0.001$.

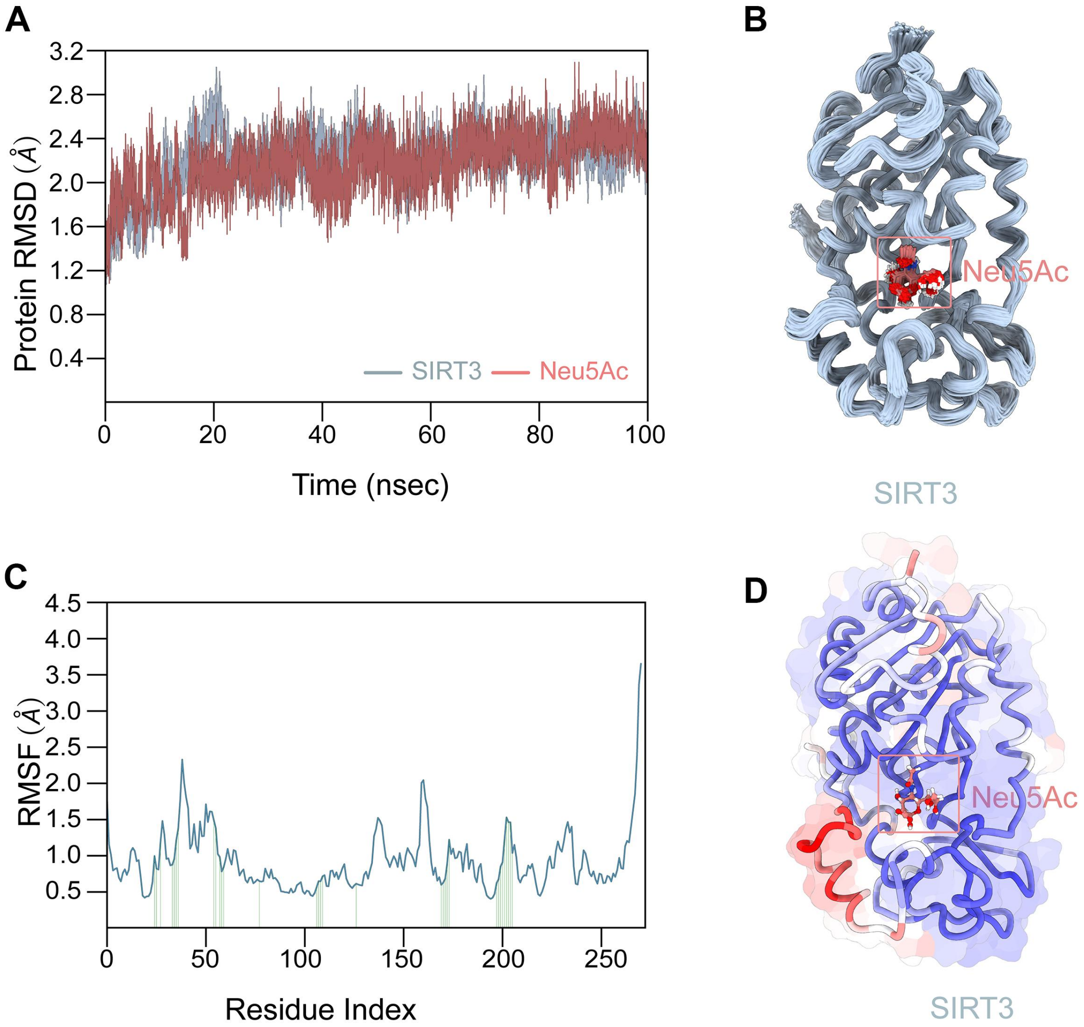

Figure S11. The binding site between Neu5Ac and SIRT3. The Neu5Ac pair was connected to SIRT3, the SIRT3-NeU5AC complex was simulated and optimized by 100ns molecular dynamics. After 100ns molecular dynamics, the molecular dynamics trajectory was analyzed: **A and B**, the RMSD of SIRT3 protein locus and Neu5Ac were extracted respectively, and the conformation of the last 10ns was extracted for superposition; **C and D**, the RMSF of the interval protein SIRT3 was calculated and the structure were colored according to the RMSF size.

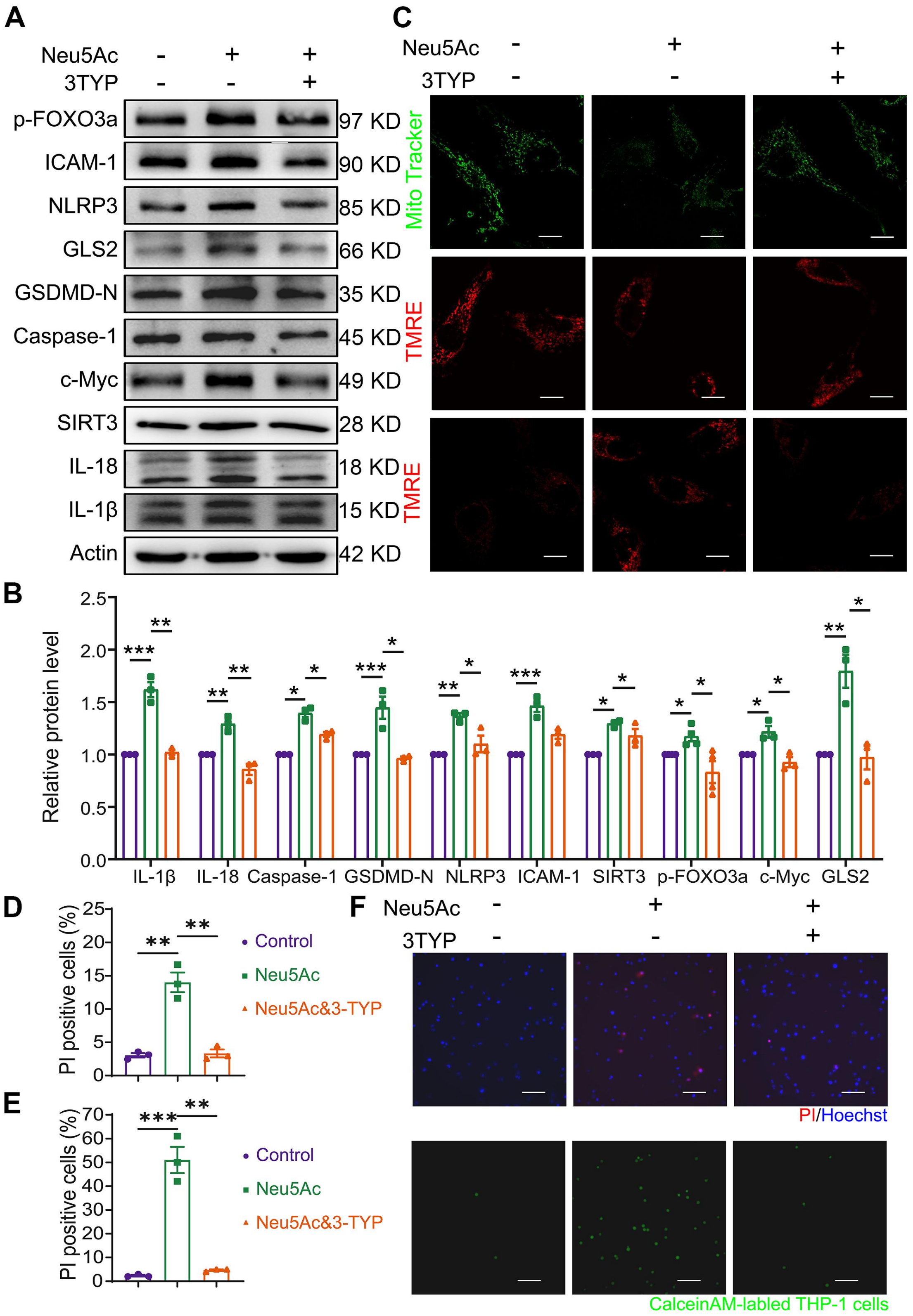

S

Figure S12. Mitochondrial damage and pyroptosis activation were alleviated in ECs after 3-YTP treatment. A-E, EA.hy926 cells were preincubated with or without

3-TYP (50 μM) for 2 h before Neu5Ac (20 mM) treatment for 12 h, IL-1β, IL-18, Caspase-1, NLRP3, GSDMD-N, GLS2 and ICAM-1 were assessed by western blot and quantified (**A and B**). **C**, mitochondrial mass, membrane potential and mitochondrial ROS production were labeled by MitoTracker Green, TMRE and mitoSOX respectively. Bar = 31.75 μm. **D**, cell death were labeled by Hoechst/PI staining and quantified. Bar = 100 μm. **E**, Monocyte-endothelial adhesion analysis were assessed and quantified. Bar = 100 μm. Data were analyzed using unpaired two-tailed student t-tests or one-way ANOVA tests, and presented as the means ± SEM. * $p < 0.05$ was considered significant, ** $p < 0.01$, *** $p < 0.001$.

Table S1

Primers used in the present study

Gene	species	Sequence (5′ → 3′)
GAPDH	Human	Forward: GCACCGTCAAGGCTGAGAAC<br>Reverse: TGGTGAAGACGCCAGTGGA
IL-1β	Human	Forward: TGGCAGAAAGGGAACAGAAA<br>Reverse: CTGGCTGATGGACAGGAGAT
IL-6	Human	Forward: GTGTGAAAGCAGCAAAGAG<br>Reverse: CTCCAAAAGACCAGTGATG
ICAM-1	Human	Forward: TCACCTATGGCAACGACTCC<br>Reverse: TCACCTATGGCAACGACTCC
ASC	Human	Forward: CTGACGGATGAGCAGTACCA<br>Reverse: CAGGATGATTTGGTGGGATT
Caspase-1	Human	Forward: CCTTAATATGCAAGACTCTCAAGGA<br>Reverse: TAAGCTGGGTTGTCCTGCACT
NLRP3	Human	Forward: CAACCTCACGTCACACTGCT<br>Reverse: TTTCAGACAACCCCAGGTTC
GLS	Human	Forward: CAGGGCAGTTTGCTTTCCAT<br>Reverse: GAGACCAGCACATCATACCCAT
GLSiso1	Human	Forward: GCAGAGGGTCATGTTGAAGTTGT<br>Reverse: GGTGTCCAAAGTGCAGTGCTT
GLSiso2	Human	Forward: ATCCTCGAAGAGAAGGTGGTGA<br>Reverse: GCAAGTTCTTGTTGGAGACTTTCA
GLS2	Human	Forward: ATCCTCGAAGAGAAGGTGGTGA<br>Reverse: ATGGCTGACAAGGCAAACCT
GLUD1	Human	Forward: TGGCATACACAATGGAGCGT<br>Reverse: TCTCAATGGCATTAACATAGGCA
GOT2	Human	Forward: CTTGAGGTTGGAGACCAGTTGAGT<br>Reverse: GATTGCTGCTGCCATTCTGA
GPT2	Human	Forward: GGCTTTGGGCAGAGGGAA<br>Reverse: TCACGCGTACTTCTCCAGGAA
MYC	Human	Forward: GGCTCCTGGCAAAAGGTCA<br>Reverse: CTGCGTAGTTGTGCTGATGT
CMYC	Human	Forward: GGGCTTTATCTAACTCGCTGTA<br>Reverse: GCTATGGGCAAAGTTTCGTG
P53	Human	Forward: GACCGGCGCACAGAGGAAGAGAATC<br>Reverse: GAGTTTTTTATGGCGGGAGGTAGAC
P63	Human	Forward: TCAGAAGATCGTGCGACAAAC<br>Reverse: GTTCAGGAGCCCCAGGTTCG
P73	Human	Forward: CTCTGGAGCTCTCTGGAACCA<br>Reverse: CGCCCACCACCTCATTATTC
GATA3	Human	Forward: AAGAGTGCCTCAAGTATCAG

		Reverse: GCGGATAGGTGGTAATGG
SIRT3	Human	Forward: CCCCAAGCCCTTTTTCACTTT<br>Reverse: CGACACTCTCTCAAGCCCA
GAPDH	Mouse	Forward: AGGTCGGTGTGAACGGATTTG<br>Reverse: GGGGTCGTTGATGGCAACA
IL-1β	Mouse	Forward: TGGACCTTCCAGGATGAGGACA<br>Reverse: GTTCATCTCGGAGCCTGTAGTG
IL-6	Mouse	Forward: ACAGAAGGAGTGGCTAAGGA<br>Reverse: AGGCATAACGCACTAGGTTT
GLS2	Mouse	Forward: TTTGCTGCATATAGTGGAGATGTC<br>Reverse: GTTGAACTGCACAGCATCGTCCAG

Table S2

siRNA sequences designed by oligobio Biotechnology, Co, Ltd (China)

	Sequence (5′ → 3′)
Human Sirt3 siRNA	CTGTGCCTAGTTGAACGGCAA
Human Gls2 siRNA 1	CGGCUAUUAUCUCAAGGAATT
Human Gls2 siRNA 2	GGAACAAGACUGUGGUCAATT
Human Gls2 siRNA 3	CCCUGGAGCCAGCUUUAAATT
