# Supplementary material for: N-Acetylneuraminic acid triggers endothelial pyroptosis and promotes atherosclerosis progression via GLS2-mediated glutaminolysis pathway
Source: Cell Death Discov. 2024 Nov 13;10:467. doi: 10.1038/s41420-024-02233-7 (PMC11561128; doi:10.1038/s41420-024-02233-7)
Supplement: Supplementary file 2 — Raw data [file 41420_2024_2233_MOESM2_ESM.pdf]

Figure 1E

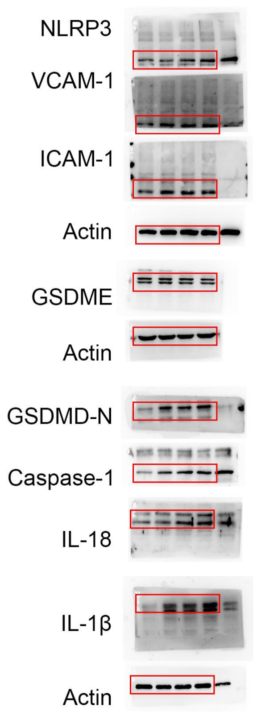

Figure 1F

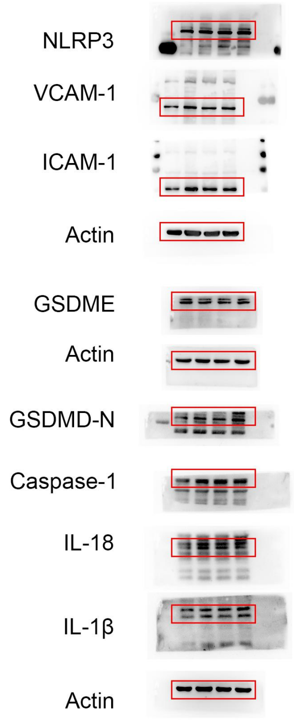

Figure 1K

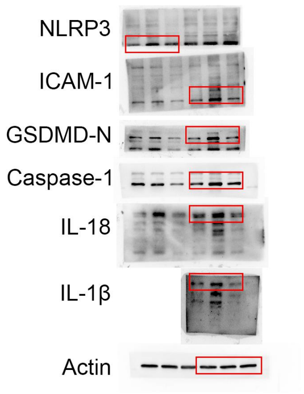

Figure 1L

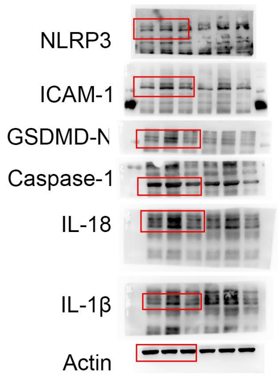

Figure 1M

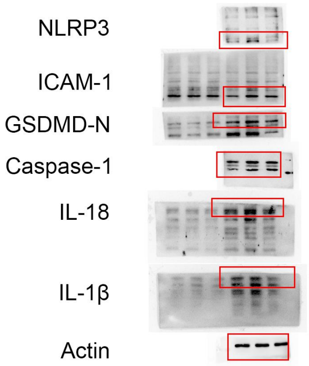

Figure 1N

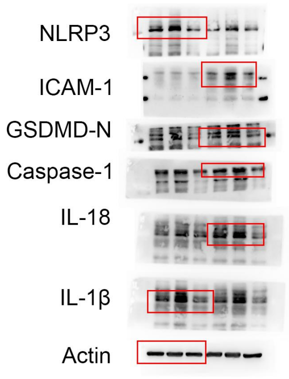

Figure 3E

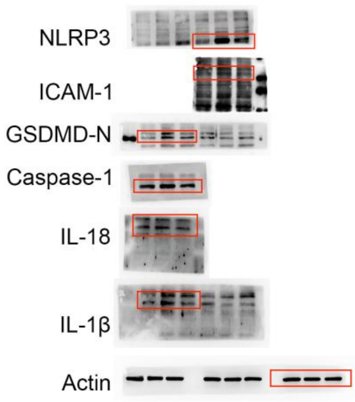

Figure 4B

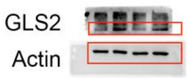

Figure 4H

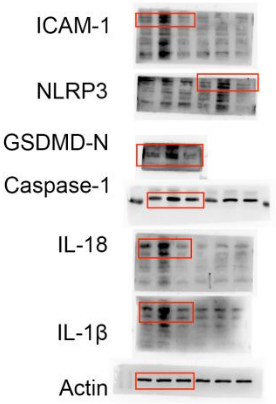

Figure 4I

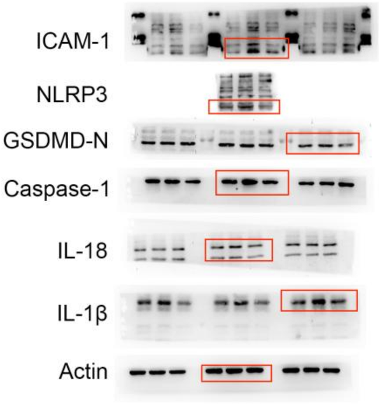

Figure 4N

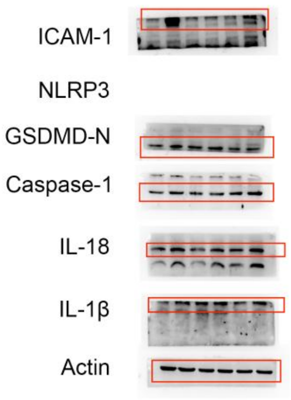

Figure 7C

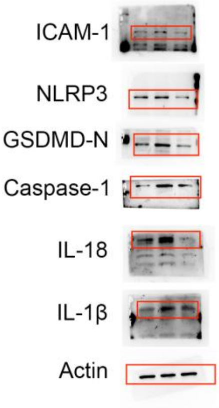

Figure 7F

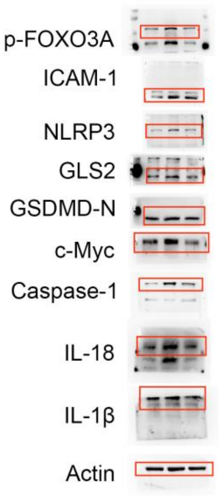

Figure 7I

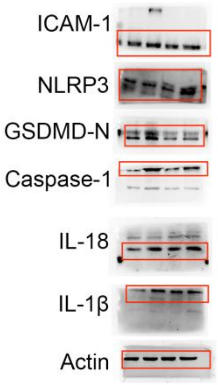

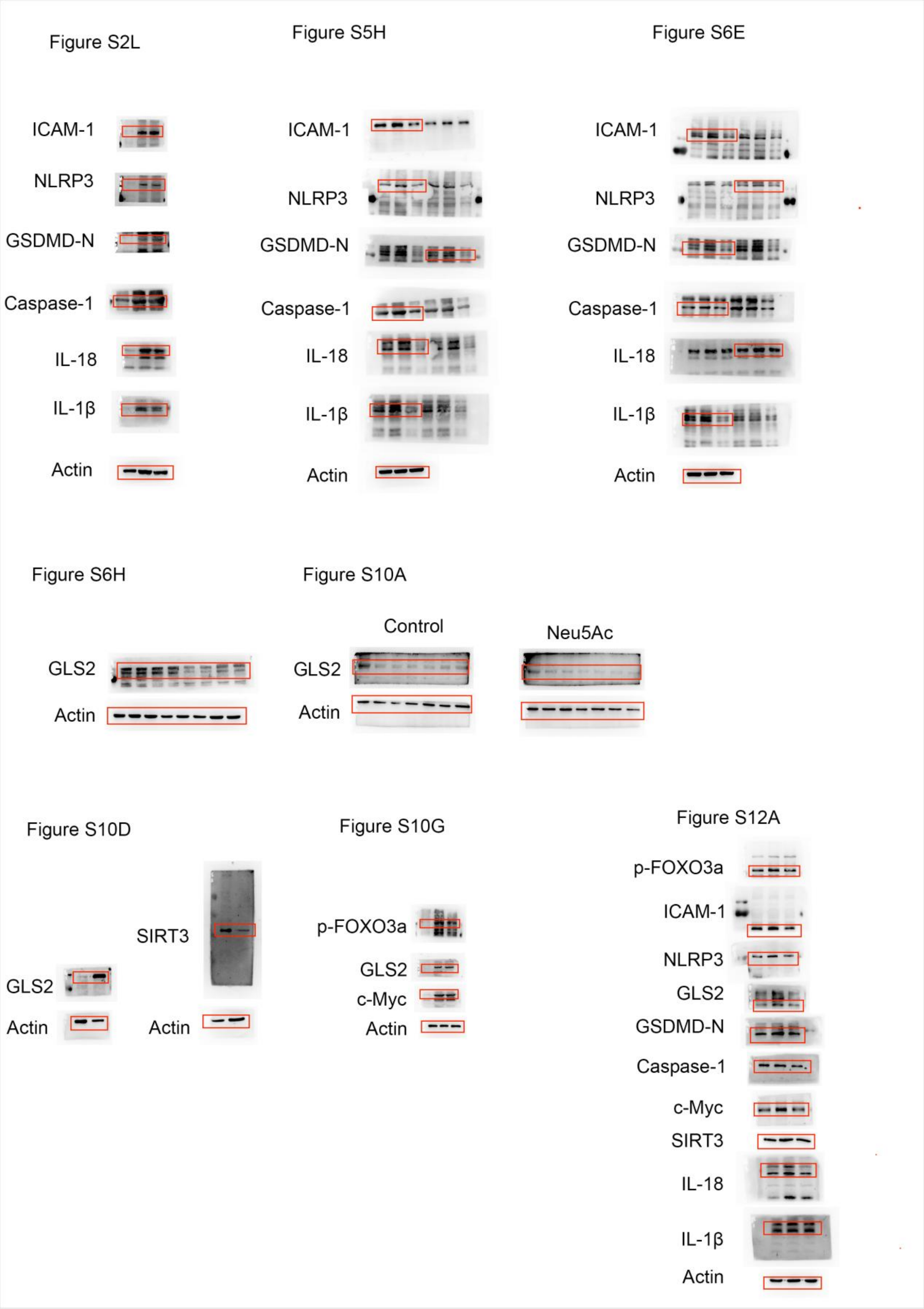
Figure S2L
ICAM-1
NLRP3
GSDMD-N
Caspase-1
IL-18
IL-1β
Actin
Figure S5H
ICAM-1
NLRP3
GSDMD-N
Caspase-1
IL-18
IL-1β
Actin
Figure S6E
ICAM-1
NLRP3
GSDMD-N
Caspase-1
IL-18
IL-1β
Actin
Figure S6H
GLS2
Actin
Figure S10A
Control
Neu5Ac
GLS2
Actin
Figure S10D
SIRT3
GLS2
Actin
Actin
Figure S10G
p-FOXO3a
GLS2
c-Myc
Actin
Figure S12A
p-FOXO3a
ICAM-1
NLRP3
GLS2
GSDMD-N
Caspase-1
c-Myc
SIRT3
IL-18
IL-1β
Actin
